# Lactic Acid Bacteria Diversity and Characterization of Probiotic Candidates in Fermented Meats

**DOI:** 10.3390/foods10071519

**Published:** 2021-07-01

**Authors:** Elvina Parlindungan, Gabriele A. Lugli, Marco Ventura, Douwe van Sinderen, Jennifer Mahony

**Affiliations:** 1 School of Microbiology & APC Microbiome Ireland, University College Cork, Western Road, T12 YT20 Cork, Ireland; elvina.parlindungan@ucc.ie; 2 Laboratory of Probiogenomics, Department of Chemistry, Life Sciences and Environmental Sustainability, University of Parma, 43121 Parma, Italy; gabrieleandrea.lugli@unipr.it (G.A.L.); marco.ventura@unipr.it (M.V.)

**Keywords:** diversity, antibiotic susceptibility, resistance, antimicrobial, antifungal, bacteriocin, organic acid, gastric juice, bile salt

## Abstract

Probiotics are defined as live microorganisms which confer health benefits to the host when administered in adequate amounts. Many lactic acid bacteria (LAB) strains have been classified as probiotics and fermented foods are an excellent source of such LAB. In this study, novel probiotic candidates from two fermented meats (pancetta and prosciutto) were isolated and characterized. LAB populations present in pancetta and prosciutto were evaluated and *Lactiplantibacillus plantarum* was found to be the dominant species. The antagonistic ability of selected isolates against LAB and non-LAB strains was investigated, in particular, the ability to produce anti-microbial compounds including organic acids and bacteriocins. Probiotic characteristics including antibiotic susceptibility, hydrophobicity and autoaggregation capacity; and ability to withstand simulated gastric juice, bile salt, phenol and NaCl were assessed. Among the characterized strains, *L. plantarum* 41G isolated from prosciutto was identified as the most robust probiotic candidate compared. Results from this study demonstrate that artisanal fermented meat is a rich source of novel strains with probiotic potential.

## 1. Introduction

With increasing public awareness for the need to improve human diet and lifestyle, there is growing market demand for functional foods and supplements which contain probiotics [1]. Probiotics are defined as live microorganisms that confer a health benefit on the host when administered in adequate amounts [2]. Lactic acid bacteria (LAB) have a long history of use as probiotics, many of which have a generally-recognized-as-safe (GRAS) status [3]. While probiotic LAB available on the market have primarily been isolated from humans [4,5], literature has identified fermented dairy, plant and meat products as a source of potentially novel probiotic LAB of extraintestinal origin [6,7,8]. Screening for novel probiotic candidates is desirable since probiotic features and health benefits conferred are known to be strain-specific [9].

Naturally fermented foods exhibit a rich biodiversity of microorganisms which make them a good source of potential probiotic LAB [10]. *Latilactobacillus sakei*, *Latilactobacillus curvatus* and *Lactiplantibacillus plantarum* are among the most prevalent LAB species associated with fermented meat products [11,12,13,14,15]. To a lesser extent, some studies have reported the presence of *Lactobacillus gasseri*, *Lactiplantibacillus pentosus*, *Lacticaseibacillus rhamnosus*, *Lactobacillus johnsonii*, *Lactococcus lactis* and *Pediococcus pentosaceus* [13,15,16]. These LAB are known to play an important role in food safety and protection through the production of antimicrobial compounds, including organic acids and bacteriocins [17,18]. The combination of different species and strains of LAB in fermented meat products has resulted in the emergence of different varieties of fermented meat products, including salami, chorizo, pancetta, prosciutto and pepperoni with an extended shelf life and unique sensory characteristics [19]. Fermented meats may be an excellent source of probiotics since they harbor high numbers of LAB [8].

Two spontaneously fermented Italian meats (pancetta and prosciutto) were acquired from a local market to screen for the presence of novel probiotic candidates. Guidelines for screening candidate probiotic LAB have been reviewed by Binda et al. [1]. Candidate probiotic strains must be (i) sufficiently characterized through evaluation of their tolerance to gastrointestinal conditions, antimicrobial activity and/or adhesion capacity; (ii) be safe, i.e., they should not be reservoirs of antibiotic resistance genes that could be transferred to pathogens; (iii) supported by at least one positive human clinical trial; and (iv) be alive in sufficient efficacious dose in the products they are applied to. This study aimed to screen for novel probiotic candidates, specifically focusing on the first two microbiological criteria.

The diversity of LAB in these two products was assessed using 16S rRNA gene-based analysis and (GTG)_5_ genetic fingerprinting. The susceptibility of the isolates to antibiotics as well as their ability to produce organic acids were investigated. Antagonistic activity of the isolates was assessed against a range of LAB and ESKAPE pathogens (*Enterococcus faecium*, *Staphylococcus aureus*, *Klebsiella pneumonia*, *Acinetobacter baumannii*, *Pseudomonas aeruginosa*, and *Enterobacter* species; highly virulent pathogens that could exhibit resistance to antibiotics) as well as fungal species. Subsequently, resistances towards bile salt, gastric juice, salt and phenol were assessed while their bile salt hydrolase (BSH) activity, autoaggregation capacity and hydrophobicity were also determined.

## 2. Materials and Methods

### 2.1. LAB Diversity, Organic Acid Profiling and Antibiotic Susceptibility

#### 2.1.1. Isolation and Growth Condition

Artisanal traditionally fermented prosciutto and pancetta were purchased from a local market in Cork, Ireland. The amount of 5 g of each sample was transferred into 45 g of phosphate buffered saline (PBS, pH 7.0) (Sigma Aldrich, St. Louis, MO, USA) and pummeled for 2 min at 300 rpm in a stomacher (Stomacher Circular 400; Seward, UK). Serial dilutions of each sample were prepared and plated on De Man, Rogosa and Sharpe (MRS) agar (Oxoid, Hampshire, UK) and incubated at 30 °C and 37 °C aerobically for 48 h. The number of colonies were counted and individual colonies were isolated for further investigation. A total of 106 individual colonies were randomly selected from pancetta (*n* = 71) and prosciutto (*n* = 35) and grown on MRS broth (Oxoid) for 24 h at 30 °C and 37 °C. Stock cultures of all isolates were stored at −80 °C in MRS broth supplemented with 30% (*v*/*v*) glycerol (Thermo Fisher, Waltham, MA, USA).

#### 2.1.2. Species Identification Using 16S rRNA Sequence Analysis

In order to identify LAB species present in pancetta and prosciutto, 16S rRNA gene amplification and sequencing were performed for 106 selected isolates using the following primers: LucF, 5′-CTTGTTACGACTTCACCC-3′ and LucR, 5′- TGCCTAATACATGCAAGT-3′ (Eurofins MWG, Ebersberg, Germany) [20]. PCR amplification of 16S rRNA genes was conducted using *Taq* DNA polymerase mastermix (Qiagen, Hilden, Germany) with the following PCR conditions: initial denaturation at 94 °C for 10 min, 30 cycles of 94 °C for 30 s, 40 °C for 30 s, 72 °C for 1 min and 30 s followed by a final extension at 72 °C for 10 min. PCR amplifications were performed with Applied Biosystems™ 2720 Thermal Cycler (Thermo Fisher). The amplicons were purified using the GenElute™ PCR Clean-Up Kit (Sigma Aldrich) according to the manufacturer’s instruction and subjected to Sanger sequencing (Eurofins MWG). The generated sequences were analyzed by comparative sequence analysis (BLASTN) against available sequence data on the National Center for Biotechnology Information (NCBI) database (https://blast.ncbi.nlm.nih.gov/Blast.cgi, accessed on 12 October 2020).

#### 2.1.3. Phylogenetic Inference

The 16S rRNA gene sequences of selected isolates were aligned and processed in EditSeq software v5.01 (DNASTAR, Inc., Madison, WI, USA). A phylogenetic tree was then constructed using the neighbour-joining method and bootstrapped employing 1000 replicates. The final tree was visualized using MEGA7 [21]. *Lactococcus lactis* L-4 (Genbank accession number: LT853603.1) was selected as an outgroup organism representing a distinct LAB species.

#### 2.1.4. Genetic Fingerprinting by (GTG)_5_ PCR

The selected isolates were subjected to PCR genomic fingerprinting with the single oligonucleotide primer (GTG)_5_, 5′-GTGGTGGTGGTGGTG-3′ [22]. PCR amplifications containing *Taq* DNA polymerase mastermix (Qiagen, Manchester, UK) were performed with Applied Biosystems™ 2720 Thermal Cycler (Thermo Fisher) with the following conditions: initial denaturation at 95 °C for 7 min; 30 cycles of 90 °C for 30 s, 40 °C for 1 min and 65 °C for 8 min; and a final extension at 65 °C for 16 min. The PCR products were applied to a 1% agarose gel containing ethidium bromide for 1 h at a constant voltage of 110 V in 1 × TAE buffer (40 mM Tris–Acetate, 1 mM EDTA, pH 8.0). The PCR profiles were visualized by UV (ultraviolet) transillumination and a digital image was captured using GeneSnap software (Syngene, MD, USA).

#### 2.1.5. Identification and Quantification of Organic Acid Production

LAB isolates were grown at the respective temperature at which they were isolated (Table S1) aerobically for 24 h. The organic acids present in the cell free supernatant (CFS) were analyzed by injecting 20 µL of the sample into an Agilent 1200 high-performance liquid chromatography (HPLC) system with a Refractive Index Detector and a REFEX 8 μ 8% H Organic Acid Column 300 × 7.8 mmol/L (Phenomenex, CA, USA). The elution fluid was H_2_SO_4_ (5 mmol/L) at a flow rate of 0.6 mL/min with the temperature of the column retained at 65 °C. The standards used were 10 mmol/L lactate, acetate, propionate, succinate and formate. MRS broth was also analyzed as a control. Statistical analysis was performed using two-way ANOVA (analysis of variance) verified with Tukey’s multiple comparison tests. A *p*-value of <0.05 was considered as statistically significant, with appropriate consideration given to samples with values close to this arbitrary threshold. All statistical analyses were performed using GraphPad Prism7 (GraphPad, CA, USA). Unless otherwise stated, all results were presented as mean ± the standard deviation of the mean of three independent experiments.

#### 2.1.6. Antibiotic Susceptibility

LAB resistance towards antibiotics was assessed by the disk diffusion method, adapted from Anisimova and Yarullina [23] with slight modifications. Briefly, isolates were grown to a concentration of 10^8^ CFU/mL. Sterile swab (Thermo Fisher) was dipped into the culture and streaked onto MRS agar plate. Disks containing antibiotics (gentamicin—10 µg/disc; vancomycin—30 µg/disc; tetracycline—30 µg/disc; ampicillin—10 µg/disc; erythromycin—15 µg/disc; rifampicin and streptomycin—10 µg/disc; penicillin—10 µg/disc; chloramphenicol—10 µg/disc; mupirocin—200 µg/disc; nalidixic acid—30 µg/disc; oxalicin—5 µg/disc) were placed on the agar using antibiotic disk dispenser (Thermo Fisher). Plates were incubated at 37 °C for 72 h. The inhibition zones (mm diameter) were scored against the following arbitrary scale: R (resistant), MS (moderately susceptible) or S (susceptible), according to established cut-off described in Table S1.

### 2.2. Antagonistic Activity

#### 2.2.1. Bacteria Indicator Strains

All indicator strains were grown in specific growth media (all supplied by Oxoid) and incubated for 24 h aerobically. They include the following: *Lactococcus cremoris* HP, MG1363, NZ9000 (M17 supplemented with 0.5% glucose (Merck, NJ, USA), 30 °C), *Leuconostoc paramesenteroides* NCDO869 (MRS, 30 °C), *Leuconostoc mesenteroides* NCDO2028 (MRS, 30 °C), *Klebsiella aerogenes* NCIMB10102 (BHI, 37 °C), *Enterococcus faecium* NCIMB11508 (BHI, 37 °C), *Pseudomonas aeruginosa* PA01 (BHI, 37 °C), *Listeria innocua* UCC3, DPC3565, DPC3566, DPC3567 (BHI, 37 °C) and *Staphylococcus aureus* NCDO949 (BHI, 37 °C).

#### 2.2.2. Spot on Lawn Antibacterial Assay

The spot-on-lawn assay was performed to evaluate the antibacterial potential of 22 selected isolates; two isolates of the same species were selected based on the source of origin and incubation conditions they were isolated from (Table S2). This method was adapted from Crowley et al. [24]. Briefly, 2 µL of 24 h grown culture isolates were spotted onto two MRS agar (Oxoid) plates, which were incubated at 37 °C for 48 h. The plates were subjected to UV treatment for 45 min. Indicator strains at concentrations of 10^5^ to 10^6^ CFU/mL were mixed with 0.8% *w*/*v* semi-solid agar and poured onto the MRS agar plates containing the isolates. The plates were incubated for a further 24 h, after which zones of inhibition surrounding the LAB colony were measured. The zones were scored against the following arbitrary scale: no inhibition observed, −; 9–15 mm zone of inhibition, +; 16–22 mm zone of inhibition, + +; 23–29 mm zone of inhibition, + + +; ≥30 mm zone of inhibition, + + + +. If the zone of inhibition was present, the ‘+’ symbol is presented whereas if the zone of inhibition was hazy, this symbol will be color-coded red. Assays were performed at least in triplicate.

#### 2.2.3. Well Diffusion Assay to Detect Bacteriocin Production

Three isolates exhibiting clear antimicrobial activity (*L. plantarum* 13A and *P. acidilactici* 40J from pancetta and *L. plantarum* 41P from prosciutto) were identified based on the spot assay and were selected for further bacteriocin activity analyses using well diffusion assays adapted from Tagg and McGiven [25]. Briefly, 24 h grown cultures of selected isolates were centrifuged at 4000× *g* for 10 min at 4 °C. The cell-free supernatant (CFS), which was the culture broth without cell pellet, was collected, filtered through 0.20 µm pore size filters (Whatman International Ltd., Maidstone, UK), neutralized to pH 7 using 5 M NaOH (Thermo Fisher) and 5 mg/mL catalase (Sigma Aldrich) was added to ascertain if pH was the inhibitory factor and to eliminate the effect of other antimicrobial compounds/factors such as H_2_O_2_. Fresh overnight cultures of each indicator strain (of 10^5^ to 10^6^ CFU/mL) were mixed with semi-solid (0.8% *w*/*v*) agar. Upon solidification, wells with a diameter of 8 mm were prepared using sterile pipette tips. The amount of 100 µL of cell free supernatant (CFS) was added into each well and left at room temperature until the supernatant diffused into the agar. The plates were then incubated at 37 °C. After 24 h, the zones of inhibition were measured and the results were recorded as ‘+’ where inhibition was observed and ‘−’ where no inhibition was observed. Assays were performed in at least triplicate.

#### 2.2.4. Spot-on-Lawn Antifungal Assay

The spot-on-lawn assay was performed to evaluate the antifungal potential of the 22 isolates by employing a method adapted from Crowley et al. [24]. Briefly, 2 µL of 24 h grown culture isolates were spotted onto MRS agar (Oxoid) plates, which were incubated at 37 °C for 48 h. Fungal strains *Penicillium digitatum* DSM2731 and *Penicillium expansum* DSM1282 were obtained from the DSMZ culture collection (Braunschweig, Germany) and were cultivated on Sabouraud 4% dextrose agar (Sigma Aldrich) at 30 °C for at least 4 days or until sporulation occurred. Fungal spore suspensions were prepared by scraping spores from the surface of the mold lawn and suspending the spores in 1/4 strength Ringer’s solution containing 0.8% Tween 80. The MRS plates were overlaid with Sabouraud 4% dextrose semi-solid agar seeded with ∼10^4^ to 10^5^ spores/mL, containing 2.5 µg/mL ampicillin (Sigma Aldrich) to retard bacterial growth. Plates were incubated at 30 °C for 48 h and the zones of inhibition surrounding the LAB colony were measured. The zones were scored against the following arbitrary scale: no inhibition observed, −; 9–15 mm zone of inhibition, +; 16–22 mm zone of inhibition, + +; 23–29 mm zone of inhibition, + + +; ≥30 mm zone of inhibition, + + + +. Assays were performed in at least triplicate. Antifungal strains *L. plantarum* 16 and 62 [24], sourced from the University College Cork (UCC) culture collection, were used as positive controls for the anti-fungal assay.

### 2.3. Screening for Probiotic Candidates

#### 2.3.1. Phenol and Salt Tolerance

Based on the results of the antibiotic resistance tests, 12 isolates (*L. curvatus* 41A, 15E and 15A; *L. sakei* 42C; *L. plantarum* 38I, 13A, 37F, 41G and 41P; *P. acidilactici* 40J; *L. coryniformis* subsp. *torquens* 42L and 14I) were selected for further analysis. Overnight cultures (1% *v*/*v*) were inoculated into MRS broth containing 0.2 or 0.5% (*v*/*v*) phenol and 1.5, 2.5 or 3.5% (*w*/*v*) sodium chloride. Phenols can inhibit the growth of LAB and thus resistance to phenol is important for their survival in the gastrointestinal tract [26]. While tolerance to NaCl is not a probiotic feature, the incorporation of probiotic LAB in food products presents major technical challenges if they are sensitive to salt present in the food products that they are added to. Phenol and NaCl tests were performed following the methods by Shehata et al. [27] and Mafra et al. [28]. MRS broth containing the isolates without phenol and sodium chloride was used as controls in the assay. Bacterial cells in the culture broth were measured by measuring the optical density (OD_600_) after 24 h incubation at 37 °C. Results were presented as relative abundance (%) according to the following formula.

% Relative abundance = ODt (test sample)/ODc (positive control) × 100%

#### 2.3.2. Bile Salt Tolerance

Bile salt tolerance of 12 LAB isolates (those referred to in the previous section) was evaluated following a method adapted from Shehata et al. [27]. In brief, fresh overnight cultures of the isolates (1% *v*/*v*) were sub-cultured in 10 mL MRS broth for 20 h at 37 °C. The cells were washed twice in saline (0.85% *w*/*v*) and resuspended in MRS broth containing 0.3% (*w*/*v*) bile salt (Sigma Aldrich) and incubated at 37 °C for 3 h. This concentration was applied as 0.3% (*w*/*v*) is believed to be the average representative of bile salt concentration in the gastrointestinal tract [29]. Aliquots of 100 µL were removed at constant intervals (t = 0, 1, 2, 3 h) and spread on MRS agar plates; plates were incubated at 37 °C for 72 h to determine total viable cell count (CFU/mL).

#### 2.3.3. Simulated Gastric Juice Tolerance

Simulated gastric juice tolerance of the 12 LAB isolates was tested, following a method adapted from Shehata et al. [27]. Briefly, overnight cultures of the isolates (1% *v*/*v*) were sub-cultured in 10 mL MRS broth for 20 h at 37 °C. The cells were washed three times with PBS (Sigma Aldrich) pH 7.0 and then resuspended in 1 mL of the same buffer. The amounts of 5 mL of simulated gastric juice (made by resuspending pepsin (3 g/L) in sterile saline (0.5% *w*/*v*), pH adjusted to 2.0) and 1.5 mL saline solution (0.5% *w*/*v*) were added to the resuspended cell pellet. The mixture was vortexed for 10 s and incubated at 37 °C for 3 h. Aliquots of 100 µL were removed at constant intervals (t = 0, 1, 2, 3 h) and spread on MRS agar plates; plates were incubated at 37 °C for 72 h to determine total viable cell count (CFU/mL) after exposure to the simulated gastric juice.

#### 2.3.4. Analysis of Autoaggregation and Hydrophobicity Properties

Autoaggregation and hydrophobicity analysis were performed using the method described by Melo et al. [30] and Vinderola et al. [31], respectively, with some modifications. Briefly, isolates were cultured in 10 mL MRS broth overnight at 37 °C. The bacterial pellet was collected and resuspended in saline (0.85% *w*/*v*) solution to an optical density (OD_600_) of 0.40. The suspension was incubated at 37 °C for 3, 5 and 24 h and the OD_600_ was monitored hourly. OD_i_ refers to initial measurement at t = 0 h; OD_f_ refers to final measurement at t = 3, 5 or 24 h. The percentage aggregation (A) was measured as described below.

% A = [ODi − ODf]/ODi × 100%.

Hexane was used to determine the hydrophobicity of the cell surface. The amount of 600 µL of hexane (RCI Labscan, Bangkok, Thailand) was added to 3 mL of cell suspension and mixed by vortexing thoroughly for 2 min. The mixtures were incubated at 37 °C for 1 h, allowing the hydrocarbon phase to rise completely. The aqueous phase was carefully removed and the hydrophobicity (H) was measured as follows.

% H = [ODi (before hexane added) − ODf (after hexane added)]/ODi × 100%

### 2.4. Bioinformatic Identification of Bacteriocin Gene Clusters and Probiotic Gene Markers

#### 2.4.1. DNA Extraction, Genome Sequencing, Assembly and Annotation

DNA was extracted from a fresh 10 mL overnight culture of selected bacteriocin producing/probiotic candidates (*L. plantarum* 13A, 41P, 41G, 38I; *P. acidilactici* 40J; *L. coryniformis* subsp. *torquens* 42L and 14I) using Invitrogen PureLink™ Genomic DNA Mini Kit (Thermo Fisher) according to the manufacturer’s instructions with some modifications. The cell pellet was resuspended and incubated in TE buffer containing 25% sucrose (Thermo Fisher) and 30 mg/mL lysozyme (Sigma Aldrich). Chromosomal DNA extracted from each strain was sequenced by the commercial sequencing service provider GenProbio srl (Parma, Italy) using an Illumina MiSeq platform. Genomic libraries were constructed using the TruSeq DNA PCR-Free LT Kit (Illumina^®^) and 2.5 μg of genomic DNA, which was fragmented with a Bioruptor NGS ultrasonicator (Diagenode, Denville, NJ, USA) followed by size evaluation using Tape Station 2200 (Agilent Technologies, Santa Clara, CA, USA). Library samples were loaded into a Flow Cell V3 600 cycles (Illumina^®^) and draft genome Illumina sequencing was performed on a MiSeq genomic platform (Illumina, Cambridge, UK) at GenProbio srl (Parma, Italy). Fastq files of the paired-end reads obtained from the genome sequencing were used as input for genome assemblies through the MEGAnnotator pipeline in default mode [32]. The MIRA program (version 4.0.2) was used for de novo assembly of genome sequence data [33]. Following final genome assembly, putative protein-encoding genes were identified using the prediction software Prodigal (version 2.0) [34]. Protein-encoding genes were automatically annotated using a BLASTP v2.2.26 (cut-off E-value of 0.0001) sequence alignment against the non-redundant protein (nr) database curated by NCBI (ftp://ftp.ncbi.nih.gov/blast/db/, accessed on 18 November 2020).

Genome sequencing of *L. plantarum* 41P was performed using a combination of the Illumina MiSeq platform as described above and the Pacific Bioscience (Pacbio) SMRT RSII sequencing platform (PacBio, Macrogen, Seoul, Korea). The obtained raw reads were assembled with the Hierarchical Genome Assembly Process (HGAP) pipeline using the protocol RS_Assembly.2 implemented in SMRT Smart Analysis portal v.2.3 (https://www.pacb.com/support/software-downloads/, accessed on 4 May 2021). In order to achieve a complete circular genome, paired reads from the Illumina platform and filtered subreads from the Pacbio platform were assembled using Unicycler v0.4.8-beta [35] in bold mode. For chromosomal contigs, overlapping regions were identified using BLASTN, trimmed and rotated so the genome commenced with *dnaA*. Automatic annotation of predicted open reading frames (ORFs) was performed using a combination of PRODIGAL v.2.6.3 (https://github.com/hyattpd/Prodigal, accessed on 4 May 2021) and BLASTP alignments to assign annotation (using an E-value cut-off of 0.0001 for hits showing at least 50% of similarity across at least 50% of the sequence length) against a non-redundant protein database provided by the National Centre for Biotechnology Information portal (http://www.ncbi.nlm.nih.gov/, accessed on 4 May 2021). Where appropriate, automatic annotation was refined with information obtained from similarity searches involving alternative databases such as protein families (Pfam) [36] and clusters of orthologous groups of proteins (COG) [37]. Ribosomal RNA (rRNA) and transfer RNA (tRNA) genes were detected using RNAMMER v1.2 (http://www.cbs.dtu.dk/services/RNAmmer/, accessed on 4 May 2021) and tRNA-scanSE v2.0 (http://lowelab.ucsc.edu/tRNAscan-SE/, accessed on 4 May 2021), respectively.

#### 2.4.2. Identification of Bacteriocin-Encoding Gene Cluster

The genome sequences of bacteriocin-producing probiotic candidates were analyzed using BAGEL4 [38] and antiSMASH [39] to identify potential loci associated with the production of bacteriocin(s). Potential areas of interest identified by BAGEL4 and antiSMASH were manually checked and compared to previously identified bacteriocin-associated operons where appropriate.

#### 2.4.3. Genotypic and Phenotypic Characterization of Probiotic Functions

The genome sequences of probiotic candidates were investigated for the presence of previously reported probiotic gene markers such as those encoding bile salt hydrolase (*bsh*), fibronectin binding protein (*fbp*) and mucin binding protein (*mub*) based on sequence homology to previously characterized equivalents available in the NCBI (ftp://ftp.ncbi.nih.gov/blast/db/, accessed on 7 May 2021) and UniProt (https://www.uniprot.org/, accessed on 7 May 2021) database. In order to phenotypically confirm BSH activity of selected LAB isolates, the isolates were grown on MRS agar plates containing 0.5% (*w*/*v*) taurodeoxycholic acid sodium salt (TDCA; Sigma Aldrich) [40] and incubated at 37 °C for 72 h. The bile conjugation activities of colonies were manifested in two forms—either through a precipitation zone formation around colonies or through the production of opaque white colonies without precipitate halos.

### 2.5. Statistical Analysis

Statistical analysis was performed using two-way ANOVA verified with Tukey’s multiple comparison tests. A *p*-value of less than 0.05 was considered as statistically significant. All statistical analyses were performed using GraphPad Prism7 (GraphPad, San Diago, CA, USA). Unless otherwise stated, all results were presented as mean ± standard deviation of three independent experiments.

### 2.6. Genbank Accession Numbers

Genbank accession numbers include the following: JAECZS000000000 (*L. plantarum* 13A), CP075330 to CP075335 (*L. plantarum* 41P), JAHBBQ000000000 (*L. plantarum* 41G), JAHBBR000000000 (*L. plantarum* 38I), JAHBBT000000000 (*L. coryniformis* subsp. *torquens* 42L), JAHBBS000000000 (*L. coryniformis* subsp. *torquens* 14I), JAECZQ000000000 (*P. acidilactici* 40J), AY705375.1 (*P. acidilactici* K10), X94434.2 (*L. plantarum* C11), NDXC01000075.1 (*L. plantarum* NI326) and DQ323671.2 (*L. plantarum* J23).

## 3. Results and Discussion

### 3.1. Diversity of LAB in Pancetta and Prosciutto

**Figure 1:**
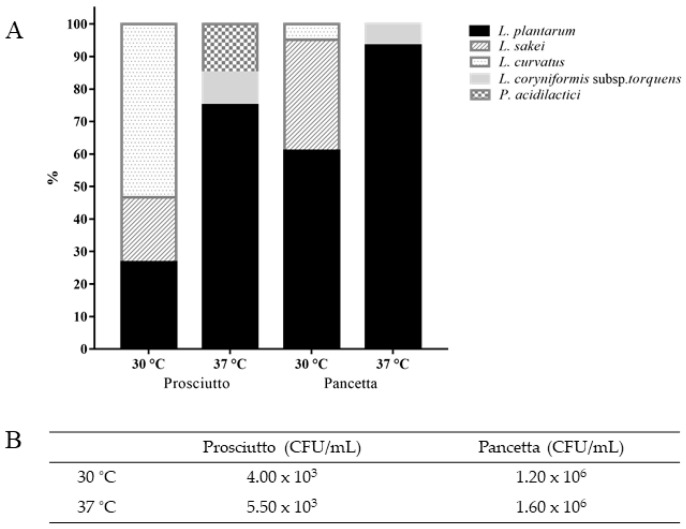
(**A**) LAB species populations identified based on 16S rRNA gene sequence analysis. A total of 35 isolates (*n* = 15 at 30 °C and *n* = 20 at 37 °C) from prosciutto and 71 isolates (*n* = 41 at 30 °C and *n* = 30 at 37 °C) were selected from pancetta. (**B**) Bacterial counts (CFU/mL) on MRS agar after 48 h incubation at 30 °C and 37 °C incubation from prosciutto and pancetta.

Fermented meats are reported to be a rich source of LAB and we therefore evaluated and compared the microbial composition of two Italian fermented meat products. This was achieved through a combination of classical microbiological and molecular approaches, permitting an evaluation of the diversity and distribution of LAB in prosciutto and pancetta. MRS was used as a selective medium to isolate potential probiotic LAB. Prior to the isolation of individual colonies, the number of viable colonies (CFU/mL) of both products was counted on MRS agar at 30 °C and 37 °C. The prosciutto sample was observed to host approximately 10^3^ CFU/mL, while significantly higher counts were observed for pancetta at approximately 10^6^ CFU/mL at both temperatures (Figure 1B). A total of 35 isolates from prosciutto and 71 isolates from pancetta were retrieved for 16S rRNA gene sequence analysis.

Analysis of the isolates derived from prosciutto at 30 °C suggests a dominance of *L. curvatus* (8/15 or 53%), in addition to *L. plantarum* (4/15 or 27%) and *L. sakei* (3/15 or 20%) (Figure 1A). At an incubation temperature of 37 °C, however, the majority of the isolated LAB population was represented by *L. plantarum* (15/20 or 75%) with a significantly lower percentage of *P. acidilactici* (3/20 or 15%) and *Loigolactobacillus coryniformis* subsp. *torquens* (2/20 or 10%). In the pancetta sample, the dominant LAB species appeared to be *L. plantarum* regardless of the incubation temperature (Figure 1A). There was a comparatively higher proportion of *L. sakei* (14/41 or 34%) compared to *L. curvatus* (2/41 or 5%) at 30 °C while a small subpopulation of *L. coryniformis* subsp. *torquens* (2/30 or 7%) was identified specifically at 37 °C. Overall, *L. plantarum* was the predominant LAB species in both meat products and prosciutto appeared to exhibit a higher diversity of organisms depending on the incubation temperature. *L. coryniformis* subsp. *torquens* and *P. acidilactici* were identified specifically at 37 °C, whereas *L. sakei* and *L. curvatus* were determined to be more dominant at 30 °C (Figure 1A). *L. plantarum* isolates were obtained at both 30 °C and 37 °C, which likely reflects this species’ versatility and adaptability to grow at different temperatures.

Long fermentation and ripening times expose LAB to prolonged stress, including acid stress, oxidation and starvation, which in turn affects their survival in specific food niches. The accumulation of lactic acid produced by LAB could exert physiological changes [41], which may cause the collapse of the proton motive forces as it acidifies the cytoplasm, resulting in cell death [42]. *L. plantarum* is regarded as one of the most versatile and industrially important LAB due to its useful properties, a high survivability and being functional in a range of fermented food niches [43]. *L. plantarum* was also observed to be among the dominant LAB species found in fermented meat products of different geographical regions, including Asia [44,45], South America [46] and Africa [47]. It is generally accepted that *L. plantarum* is highly tolerant against acid and alkali stress and some strains of *L. plantarum* were reported to be able to withstand heat, oxidative strees and starvation stress [48,49,50].

A previous study of LAB diversity in fermented meats produced in a range of European countries highlighted a dominance of *L. sakei* in a variety of fermented meat products [51]. The second most predominant species was *P. pentosaceus* (for Belgium and Germany) or *L. curvatus* (for Spain, France and Italy). *P. acidilactici* and *L. coryniformis* subps. *torquens* species have also been found in fermented meat products [52,53,54,55], although they were not commonly identified as the dominant LAB species surviving in European fermented meats. The findings in the present study concur with what has been reported in literature as Figure 1A demonstrated low percentage of *L. coryniformis* subps. *torquens* (10% in prosciutto and 5% in pancetta) and *P. acidilactici* (15% in prosciutto). However, it is noteworthy that the dominant LAB components identified in the present study differed from those observed in the pan-European study, suggesting a product-specific microflora.

**Figure 2:**
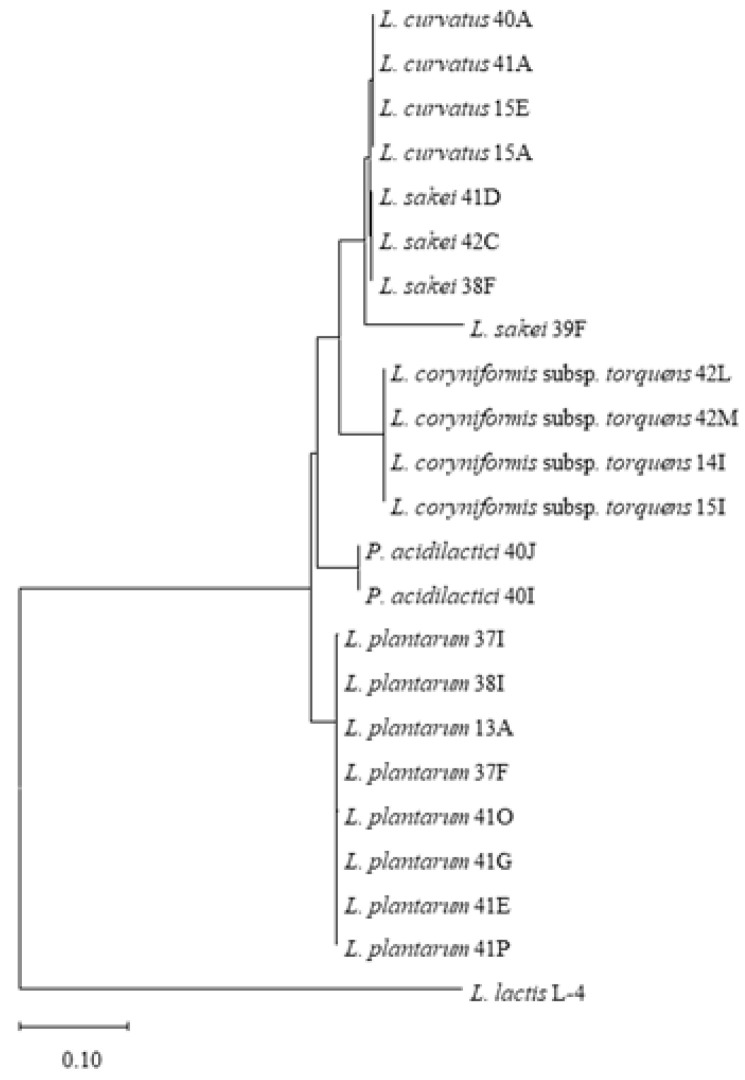
Phylogenetic tree based on the 16S rRNA gene sequences of 22 selected LAB species. *Lactococcus lactis* L-4 was applied as the outgroup.

Based on the 16S rRNA gene sequence data, food source and incubation temperature (Table S2), we selected 22 representative LAB isolates and subjected these strains to genetic fingerprinting analysis (GTG)_5_ (Figure S1), thereby yielding species level profiles which confirmed the 16S rRNA speciation results of the selected isolates. Isolates of *L. plantarum* (lanes 1–8), *L. coryniformis* subsp. *torquens* (lanes 17–20) and *P. acidilactici* (lanes 21–22) were shown to exhibit species-specific profiles. Furthermore, (GTG)_5_ profiles of *L. sakei* strains 38F and 39F (lanes 11–12) were shown to be highly similar while that of *L. sakei* 41D was shown to lack a band (~1400 bp), whereas the *L. sakei* 42C strain produced a slightly smaller band of less than 1400 bp, suggesting that these may be distinct isolates. While the genetic fingerprinting profiles of *L. curvatus* 40A, 41A and 15E (lane 13, 14 and 16) were shown to be highly similar, *L. curvatus* 15A exhibits an additional distinct amplicon (~5000 bp). The GTG species level profiling was consistent with phylogenetic analysis of the 16S rRNA gene sequences of these strains with four distinct clusters representing the five LAB species (*L. sakei* and *L. curvatus* cluster together in the phylogenetic tree) identified in this study (Figure 2 and Figure S1).

### 3.2. Organic Acid Production and Antagonistic Activity

**Figure 3:**
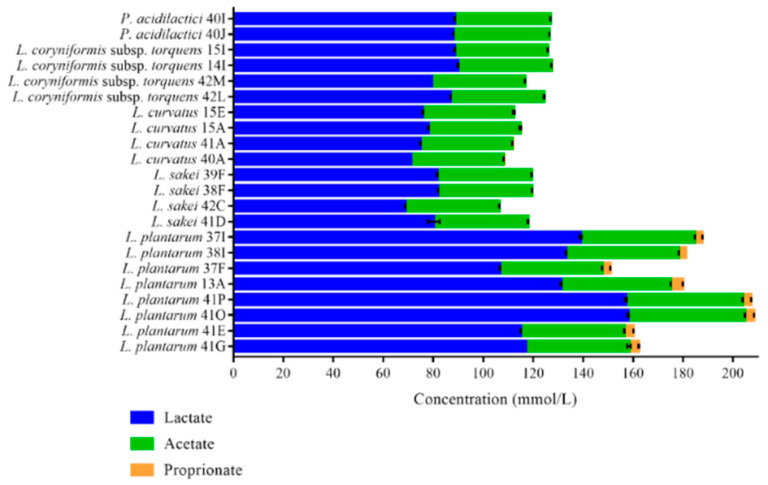
Concentration of organic acids (mmol/L) in the CFS produced by 22 selected LAB species. The data indicates mean ± standard deviation of three independent experiments.

LAB may possess a competitive advantage to survive through the production of antimicrobial compounds including organic acids. LAB may produce more than one type of organic acid (although primarily lactic acid) and this may vary by species and even strain. In order to evaluate the organic acid production profiles of 22 representative isolates, the cell-free supernatant (CFS) of fresh overnight cultures of the selected strains were analyzed by HPLC. Representative isolates of *L. plantarum*, *L. sakei*, *L. curvatus*, *L. coryniformis* subsp. *torquens* and *P. acidilactici* were analyzed in this manner. Perhaps unsurprisingly, the isolates were observed to primarily produce lactic and acetic acids (Figure 3, Table S3). Among the five different species of LAB, strains of *L. plantarum* produced the highest concentration of lactic acid compared to other species, with *L. plantarum* 41O and 41P producing significantly (*p* < 0.001) higher lactate of 157.09 ± 0.29 mmol/L and 158.02 ± 0.22 mmol/L, respectively, compared to *L. plantarum* 41G, 41E, 13A, 37F, 38I and 37I (106.67 to 138.91 mmol/L). *L. curvatus* (71.14 to 78.07 mmol/L) and *L. sakei* (68.75 to 81.90 mmol/L) were significantly weaker lactic acid producers compared to *P. acidilactici* (~88 mM). In general, *L. plantarum* species also formed significantly (*p* < 0.001) higher acetate compared to other species, with *L. plantarum* 41O, 41P, 37I and 38I (ranging from 45.16 to 46.77 mmol/L) being the strongest than compared to *L. plantarum* 41G, 41E, 13A and 37F (ranging from 40.94 to 43.82 mmol/L). The concentration of acetate produced by *L. sakei* did not significantly (*p* > 0.05) differ compared to *L. coryniformis* subsp. *torquens*, *P. acidilactici* and *L. curvatus*. Furthermore, propionate was not produced by the tested isolates except for *L. plantarum* at a range of 2.81 to 4.90 mmol/L.

The inhibitory activity of the selected 22 LAB isolates was assessed using the spot-on-lawn antimicrobial assay against several indicator organisms associated with food spoilage and/or food poisoning. The inhibitory activity of LAB may be due to the production of organic acids and/or bacteriocins and thus it was aimed to discern the source of antimicrobial activity where relevant. The nature of inhibitory activity was classified into two types (shown in Figure S2): If a hazy undefined zone was observed, then the inhibitory effect was predicted to be primarily due to organic acid production, whereas if a clear defined zone was observed it was presumed to be due to bacteriocins [56]. In summary, all *L. plantarum* and *L. sakei* isolates exhibited inhibitory activity against all indicators tested in the spot-on-lawn assays (*L. cremoris* HP; *L. paramesenteroides* NCDO869; *L. mesenteroides* NCDO2028; *K. aerogenes* NCIMB10102; *E. faecium* NCIMB11508; *P. aeruginosa* PA01; *L. innocua* UCC3 and *S. aureus* NCDO949) (Table 1). *L. curvatus* 40A and 41A exhibited weak inhibitory activity against *L. cremoris* HP and *L. curvatus* 15A and 15E presented a weak antagonistic effect against *S. aureus* NCDO944 alone among the tested indicator strains. *L. coryniformis* subsp. *torquens* 42L and 42M demonstrated weak to moderate inhibitory activity against all indicators, whereas 14I and 15I showed similar activity against all indicators except for *L. cremoris* HP and *E. aerogenes* NCIMB10172 (Table 1). *L. innocua* UCC3, *E. faecium* NCIMB11508 and *S. aureus* NCDO944 were strongly inhibited by *P. acidilactici* 40J and 40I (Table 1).

The effectiveness of organic acids as a natural preservative in contributing to food protection and safety against a range of spoilage and pathogenic microorganisms has been well studied [57,58,59]. Bacterial genera associated with food spoilage, such as *Lactococcus*, *Leuconostoc*, *Klebsiella* and *Pseudomonas* [60,61], and pathogenic bacteria associated with food poisoning, such as *Listeria* and *Staphylococcus* [62,63], were used as indicators in this study (Table 1). Among the 22 strains tested, 14 isolates of *L. plantarum*, *L. sakei* and *P. acidilactici* inhibited all indicators tested, whereas *L. curvatus* and *L. coryniformis* subsp. *torquens* showed antagonistic ability at least against one indicator tested. Additionally, the antifungal potential of the isolates was evaluated. All isolates of *L. plantarum*, *P. acidilactici* and *L. coryniformis* subsp. *torquens* inhibited *P. expansum* DSM1282 (Table 1), but none of the isolates were active against *P. digitatum* DSM2731. *P. expansum* is recognized as one of the most dominant post-harvest pathogens in fruits and vegetables mainly in pomaceous fruits [64]. While the compound(s) that exert the anti-fungal activity are not defined within the scope of this study, previous studies have reported the anti-fungal activity of phenyllactic acid produced by *L. plantarum* [65] and a bacteriocin-like compound produced by *P. pentosaceous* [66].

Findings from this study indicate that the presence of LAB in prosciutto and pancetta play a crucial role in controlling spoilage and/or pathogenic microorganisms through the production of organic acids and, in some cases, bacteriocins. However, it is important to note that organic acids and bacteriocins may not be the sole contributing factors, as LAB may also produce a variety of antimicrobial compounds including hydrogen peroxide, carboxylic acid, diacetyl and reuterin [67].

### 3.3. Bacteriocin Activity and Associated Gene Clusters

In order to further validate that the inhibitory effect exerted by LAB isolates were not due to the reduced pH, well diffusion assays using pH neutralized and catalase treated (to remove H_2_O_2_ effect) CFS from selected representative isolates—*L. plantarum* 13A, 41G, 38I, 41P and *P. acidilactici* 40J (Table 2)—were performed. These representative isolates were selected based on the product origin and the incubation temperature they were isolated from, as shown in Table S2. These assays confirmed that *L. plantarum* 13A, 41G, 38I and 41P produced bacteriocins that were effective against *L. cremoris* HP, MG1363, NZ9000 and *L. paramesenteroides* NCDO869, while that produced by *P. acidilactici* 40J was effective against *L. mesenteroides* NCDO2028, *E. faecium* NCIMB11508 and *L. innocua* strains UCC3, DPC3565, DPC3566 and DPC3567 (Table 2). Growth of *K. aerogenes* NCIMB10102, *P. aeruginosa* PA01 and *S. aureus* NCDO949 was not inhibited by the three tested isolates, which suggests that the inhibitory activity against these indicators was due to organic acids (Table 1). In addition, the compounds produced by *L. plantarum* 13A, 41G, 38I, 41P and *P. acidilactici* 40J were sensitive to proteinase K, which further substantiated their proteinaceous nature.

In order to identify bacteriocin-encoding gene clusters responsible for the observed antimicrobial activity, the genomes of five isolates were sequenced and assembled. Potential bacteriocin gene clusters in these five isolates were detected based on in silico analysis of retrieved contigs with BAGEL4 and antiSMASH. Based on this analysis, it is proposed that *P. acidilactici* 40J produces a pediocin-like bacteriocin with genes encoding the putative structural peptide (PedA); the immunity protein PedB; the transport proteins, PedC and PedD with 99 to 100% identity to those that encode pediocin PA1 produced by *P. acidilactici* K10 (Figure S3). Additionally, the organization of the gene clusters in *P. acidilactici* 40J and K10 are identical. The bacteriocin produced by this strain (40J), which is likely pediocin, was effective against *Listeria*, *Enterococcus* and *Leuconostoc* (Table 2) and in agreement with the literature pertaining to pediocin PA1 [68].

Different strains of *L. plantarum* have been reported to produce bacteriocins with varied structural properties and antagonistic activities. For instance, *L. plantarum* B21 was previously demonstrated to be effective against a broad range of LAB and a few non-LAB species (*Listeria monocytogenes* and *Clostridium perfringens*) [69], whereas *L. plantarum* LPL-1 was effective against a broad range of non-LAB species (*L. monocytogenes*, *S. aureus*, *E. faecalis*, *B. amyloliquefaciens* and *B. pumilus)* and several strains of *Lactococcus* and *Lactobacillus* [70]. *L. plantarum* NI326 produces the cyclic bacteriocin plantaricyclin A, which shared 99 to 100% similarity to the bacteriocin encoding cluster of *L. plantarum* 13A, 38I and 41G isolated in the present study (Figure S3), that is effective against some strains of *Lactococcus*, *L. bulgaricus* and *Alicyclobacillus acidoterrestris* but is not effective against *L. plantarum*, *L. brevis*, *Pediococcus*, *Listeria*, *Escherichia*, *Staphylococcus*, *Streptococcus*, *Klebsiella* and *Bacillus* [71]. Based on comparative analysis of the identified bacteriocin encoding gene clusters, it is likely that 41P produces novel bacteriocin(s), which is potentially a derivative of plantaricyclin A since the only identifiable bacteriocin-encoding region in the strain’s genome (and which is plasmid-encoded) possessed only 42% similarity to plantaricyclin A. However, further investigations through purification and identification using mass spectrometry are required to confirm its potential novelty.

### 3.4. Antibiotic Susceptibility and Screening for Probiotic Candidates

Horizontal transfer of antibiotic resistance genes, particularly those within mobile genetic elements (transposons and plasmids) deserve particular attention due to the risk of resistance transfer to pathogenic organisms [72]. The determination and comparison of antibiotic susceptibility patterns in a number of representative strains of each species are major steps in differentiating between intrinsic and acquired antibiotic resistance in probiotic bacteria [72]. Antibiotic resistance tests were first performed as a preliminary screen for potential LAB strains amongst the 22 isolates (Table 3). It is generally known that *Lactobacillus* sp. are intrinsically resistant to gentamicin, streptomycin, nalidixic acid and vancomycin [73,74]. *L. curvatus* 40A displayed widespread antibiotic-resistance amongst the *L. curvatus* strains tested, while 42C was the most sensitive amongst the *L. sakei* isolates. *L. plantarum* isolates 37F and 41G were the most antibiotic-sensitive; 37I, 38I and 41P were moderately sensitive, while 13A, 41O and 41E were resistant to several antibiotics. Nonetheless, 37F, 41G, 38I, 41P and 13A, which exhibited distinct sensitivity/resistance profiles, were selected for further probiotic tests since all *L. plantarum* isolates were bacteriocin producers (Table 1). Additionally, *P. acidilactici* 40J, *L. coryniformis* subsp. *torquens* 42L, 42M and 14I were selected for further analysis.

In this study, hexane was used to evaluate hydrophobicity of LAB cell surface. This method is considered important in determining probiotic bacterial adhesion capacity to epithelial host cells [5,75]. *L. coryniformis* subsp. *torquens* and *L. plantarum* displayed significantly (*p* < 0.05) greater % hydrophobicity (ranging from 31.2 to 42.2%) compared to *L. curvatus*, *L. sakei* and *P. acidilactici* (ranging from 4.9 to 18.1%) (Table 4). Isolates 42L, 14I, 41P, 41G and 38I did not differ significantly (*p* > 0.05) and showed highest overall % hydrophobicity. According to the classification proposed by Colloca et al. [76], bacterial hydrophobicity can be considered low (0 to 34%), moderate (35 to 69%) or high (70 to 100%). Altogether, the hydrophobicity values for meat isolates could be considered moderate when compared to those previously reported.

The auto-aggregation capacity of bacteria has also been associated with higher adhesion to epithelial cells and persistence in the intestine [77]. All isolates tested in this study were shown to exhibit auto-aggregation during the first 3 h (from 9.7 to 15.6%), which increased with time after 24 h (from 28.9 to 69.0%) (Table 4). In particular, *L. plantarum* 41P, 38I, 37F and 41G did not significantly (*p* > 0.05) differ and showed the highest auto-aggregation levels (63.6 ± 2.2 to 69.0 ± 4.2%, t = 24 h) compared to all other isolates. Autoaggregation % of *L. plantarum* strains reported in this study was similar to several lactobacilli and *L. plantarum* strains reported by other studies [77,78,79]. Thus, autoaggregation levels of meat isolates in this study are generally similar or slightly higher to the probiotic candidates reported in the literature.

The abilities to tolerate gastric juice (stomach phase) and bile salt (intestinal phase) are important properties of probiotics to ensure their viability and survival in the human gastrointestinal tract [80]. It was revealed that *L. curvatus* and *L. sakei* do not survive simulated gastric juice conditions after 1 h incubation and *L. coryniformis* subsp. *torquens* is unable to withstand such conditions following 2 h exposure (Table 5). However, they are able to survive 0.3% bile salt exposure for a period of 3 h with approximately 5-fold to 9-fold viable count reduction (Table 6). In contrast, *L. plantarum* is able to withstand simulated gastric juice to some degree with 10-fold to 15-fold reduction after 1 h, but with declined survival (ranging from 10^4^ to 10^2^ CFU/mL) after 2 h and 3 h (Table 5). These isolates appear to be more robust than previous reports on this species [78]. Similarly, *L. plantarum* strains isolated in this study were highly tolerant to bile salt stress, remainig viable at 10^8^ CFU/mL (5 to 6-fold reduction) after 3 h (Table 6). Furthermore, *P. acidilactici* remained viable at 10^5^ to 10^4^ CFU/mL after a challenge with simulated gastric juice for 1 h to 3 h (Table 5) and an 8-fold reduction in survival was observed after a challenge with bile salt for 3 h (Table 6). It was reported that the median of gastric emptying half time in healthy adults is approximately 1 h [81], but there are considerable variabilities among individuals [82]. Therefore, *L. coryniformis* subsp. *torquens* could still be considered as a probiotic candidate. In summation, all isolates display high tolerance to 0.3% bile salt (displayed by less than 10-fold reduction in survival after 3 h), but only *L. plantarum* and *P. acidilactici* strains are able to survive more than 2 h exposure to simulated gastric juice.

**Figure 4:**
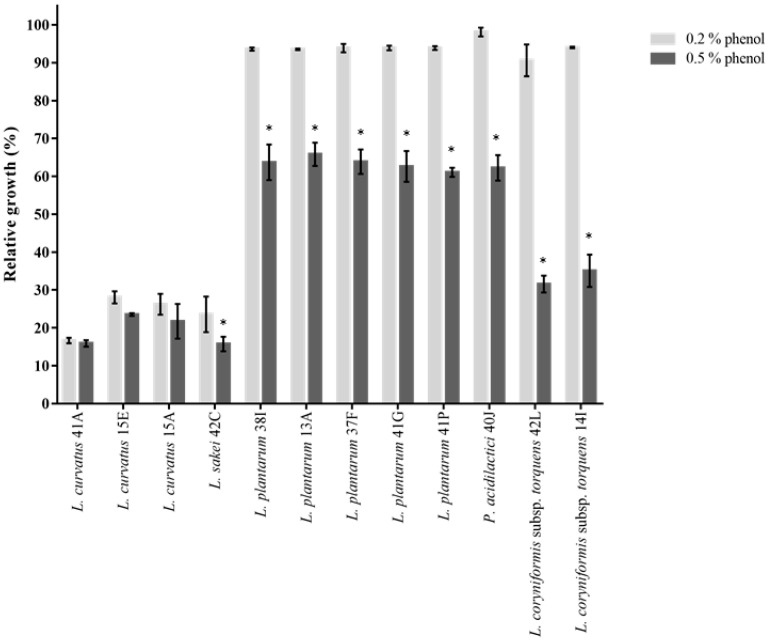
Effect of phenol on the growth of LAB isolates. Asterix (*) showed significantly different (*p* < 0.05) mean ± SD (of three independent experiments) compared to lower phenol concentration (0.2%).

Probiotics also need to survive toxic metabolites, particularly phenols produced during the digestion process. Amino acids derived from the diet or endogenous proteins can be deaminated by bacteria in the gastrointestinal tract which results in the formation of phenols [26]. All strains of *L. plantarum*, *P. acidilactici* and *L. coryniformis* subsp. *torquens* generally exhibited almost unimpaired growth in the presence of 0.2% phenol (>90% growth compared to untreated control) (Figure 4). Upon exposure to 0.5% phenol, their growth was significantly (*p* < 0.05) decreased from 61 to 65% (for 38I, 13A, 37F, 41G and 41P), 62% (for 40J), 31 and 35% (for 42L and 14I, respectively). These values were arguably higher compared to what was reported in other studies [27,78]. *L. curvatus* and *L. sakei* were nonetheless shown to exhibit low survival to 0.2% phenol (from 16 to 28%) and 0.5% phenol (from 15 to 23%) stress (Figure 4). It is important to note that phenol content in foods (ranging from flour, oil, raw beans, cocoa, fruits and wine) greatly varies from 0.0002 to 3.6% (*w*/*w*) [83]. Furthermore, its bioavailability in the human gut greatly varies depending on several factors, such as the individual’s diet, the matrix of polyphenol-rich foods which may affect intestinal absorption and, likewise, the interaction of phenols with nutrients taken from the same meal could result in alteration in their absorption [83]. For these reasons, 0.2% and 0.5% phenol concentrations (selected as the average representative phenol content present in foods) were tested against a panel of probiotic candidates.

**Figure 5:**
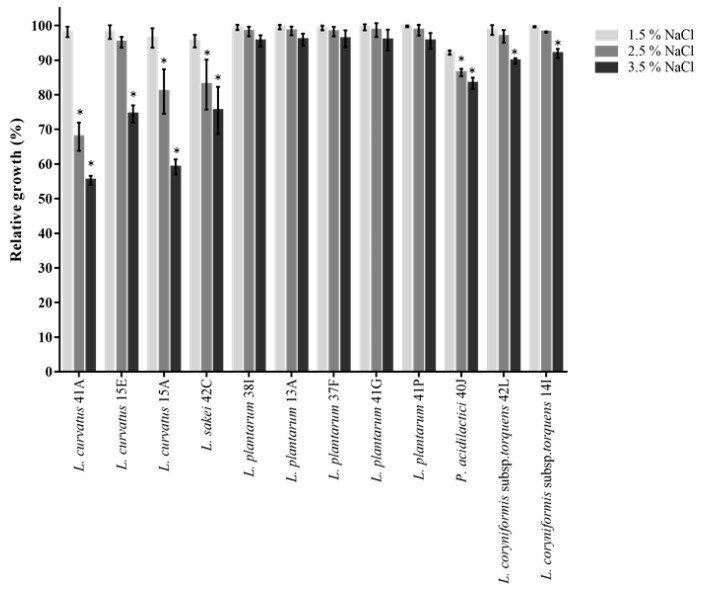
Effect of NaCl on the growth of LAB isolates. Asterisk (*) indicates significantly different (*p* < 0.05) mean ± SD (of three independent experiments) compared to lower NaCl concentration (1.5%).

During industrial processing, probiotic LAB may encounter osmotic stress due to changes in solute concentration in the environment, which could result in cellular hydration and dehydration and this may negatively affect the survival rate and influence metabolic activities of probiotics [84]. Probiotic strains could be added to fermented foods containing varying concentrations of salt. Thus, tolerance to NaCl may offer a competitive advantage for probiotics. For example, spontaneous sauerkraut fermentations operate optimally with a concentration of 2.5% (*w*/*w*) NaCl [85], while 2 to 3% salt in meat fermentation was demonstrated to improve meat texture [86]. All tested isolates were resistant to 1.5% NaCl, with *L. plantarum* being the most resistant to higher NaCl concentrations (Figure 5). Relatively high tolerance to 2.5% and 3.5% NaCl exposure was also observed for all isolates of *L. coryniformis* subsp. *torquens* (89 to 98%), *P. acidilactici* 40J (83 to 86%) and *L. curvatus* 15E (74 to 95%). Overall, *L. curvatus* 41A, 15A and *L. sakei* 42C demonstrated moderate tolerance to 2.5% (67 to 83% growth) and 3.5% (55 to 75% growth).

Current literature asserts that BSH activity offers a selective advantage for probiotic strains to survive and perform in the intestinal milieu [87]. However, it is debatable whether BSH activity provides desirable effects for the human host, as there is concern over the safety of administering BSH-positive probiotics. However, research has shown that in the case of bifidobacteria and lactobacilli, BSH-positive probiotics may not be able to dehydroxylate deconjugated bile salts and thus the majority of products resulting from BSH activity may be precipitated and excreted in feces [87]. BSH activity was assessed on selected probiotic candidates in the present study by growing them on MRS agar containing 0.5% TDCA and it was found that strains 38I, 41G, 42L and 14I produced BSH and *bsh* gene homologues were identified in the genome of these strains (Table S4). However, a *bsh* gene homologue was also identified in the genome of *P. acidilactici* 40J, which indicates that there could be a low level of BSH expression under the circumstances the bacteria were grown in or that the plate assay containing TDCA might not be the most appropriate method to test BSH activity in this strain. Other studies suggested the use of thin layer chromatography (TLC) assay as a more sensitive qualitative method to identify BSH-positive LAB [88]. The presence of other probiotic gene markers such as *mub* (mucin binding protein) and *fbp* (fibronectin binding protein), which are known to play a role in promoting probiotic adherence to the host gastrointestinal tract, was also investigated [89]. Strains 38I, 41G, 40J, 42L and 14I were found to possess homologues of the *mub* and *fbp* genes (Table S4).

## 4. Conclusions

The probiotics market is expected to grow at a rate of 5.6% annually from 2020 to 2027, reaching an estimated economic value of USD 75.9 billion by 2027 [90]. This growth is attributed to increasing public awareness regarding the health benefits of probiotics that are known to be strain-specific [91]. These probiotics could aid in the prevention of antibiotic-associated diarrhea, *Clostridium difficile* infection, nosocomial infection and travelers’ diarrhea and could also help in the treatment of inflammatory bowel syndrome, pediatric acute diarrhea and *Helicobacter pylori* infection [91]. With the increasing trend of public interest towards probiotics, it becomes crucial to characterize and discover novel probiotic strains. In this study, probiotic candidates sourced from fermented meats were assessed and identified, with *L. plantarum* 41G being the strongest candidate, considering all factors assessed in this study. Overall, this strain was the most susceptible to antibiotics (compared to other isolates belonging to the same species), with moderate hydrophobicity and auto-aggregation value, high tolerance to bile salt, phenol and NaCl stress, while it was also able to survive simulated gastric juice conditions for 2 to 3 h incubation. Additionally, but to a lesser extent, *P. acidilactici* 40J and *L. coryniformis* subsp. *torquens* 4L may be considered good probiotic candidates since the former showed inhibitory activity due to bacteriocin production, which was mainly targeted against *Listeria*, whereas the latter demonstrated moderate hydrophobicity and auto-aggregation ability similar to *L. plantarum* strains; both candidates exhibited high survivability to bile salt and NaCl stress and phenol tolerance. Nevertheless, further experiments utilizing microencapsulation are needed to explore their application potential. Finally, *L. plantarum* 41P was identified to produce a novel bacteriocin(s), while *L. plantarum*, *P. acidilactici* and *L. coryniformis* subsp. *torquens* isolates demonstrated antifungal capacity. The identification of a potentially novel antimicrobial(s)-producing strain combined with the broad anti-fungal and anti-bacterial activity of strains isolated in this study highlights the untapped potential of artisanal meat products as a source of novel probiotic strains.

## Figures and Tables

**Table 1:** Spot-on-lawn antimicrobial and antifungal assays of LAB isolates against a range of indicators: *L. cremoris* HP; *L. paramesenteroides* NCDO869; *L. mesenteroides* NCDO2028; *K. aerogenes* NCIMB10102; *E. faecium* NCIMB11508; *P. aeruginosa* PA01; *L. innocua* UCC3; *S. aureus* NCDO949; and fungal strain *P. expansum* DSM1282. + = zone of inhibition present (color-coded red if the zone of inhibition against bacteria indicators looked hazy); − = no zone of inhibition.

	*L. cremoris* HP	*L. paramesenteroides* NCDO869	*L. mesenteroides* NCDO2028	*K. aerogenes* NCIMB10102	*E. faecium* NCIMB11508	*P. aeruginosa* PA01	*L. innocua* UCC3	*S. aureus* NCDO949	*P. expansum* DSM1282
***L. plantarum* 41G**	+ + +	+ +	+	+	+	+	+ +	+	+ + +
***L. plantarum* 41E**	+ + +	+ +	+	+	+	+	+ + +	+	+ + +
***L. plantarum* 41O**	+ + +	+ +	+	+	+	+	+ + + +	+ + + +	+ + +
***L. plantarum* 41P**	+ + +	+ +	+	+ +	+	+	+ + +	+ + +	+ + +
***L. plantarum* 13A**	+ + +	+ +	+	+	+ + +	+ +	+ + +	+ + +	+ + +
***L. plantarum* 37F**	+ + +	+ +	+	+	+ + +	+	+ +	+ + +	+ +
***L. plantarum* 38I**	+ + +	+ +	+	+ +	+ +	+ + +	+ + + +	+ + +	+ + +
***L. plantarum* 37I**	+ + +	+ +	+	+ +	+ +	+ +	+ + +	+ + +	+ +
***L. sakei* 41D**	+	+	+	+	+	+	+ +	+	−
***L. sakei* 42C**	+	+	+	+	+	+	+ +	+	−
***L. sakei* 38F**	+	+	+	+	+ +	+	+ + +	+ +	−
***L. sakei* 39F**	+	+	+	+	+	+	+ + +	+ +	−
***L. curvatus* 40A**	+	−	−	−	−	−	−	−	−
***L. curvatus* 41A**	+	−	−	−	−	−	−	−	−
***L. curvatus* 15A**	−	−	−	−	−	−	−	+	−
***L. curvatus* 15E**	−	−	−	−	−	−	−	+	−
***L. coryniformis* subsp. *torquens* 42L**	+ +	−	−	+	+	+	+ +	+ +	+
***L. coryniformis* subsp. *torquens* 42M**	+	−	−	+	+ +	+	+ +	+ + +	+
***L. coryniformis* subsp. *torquens* 14I**	−	−	−	−	+	+	+	+	+
***L. coryniformis* subsp. *torquens* 15I**	−	−	−	−	+	+ +	+ +	+ +	+
***P. acidilactici* 40J**	+	−	+ + + +	+	+ + + +	+	+ + + +	+ + + +	+ +
***P. acidilactici* 40I**	+	−	+ + + +	+ +	+ + + +	+	+ + + +	+ + +	+ +

**Table 2:** Well diffusion assay of CFS (pH neutralized to 6–7, catalase added to eliminate the effect of H_2_O_2_) of *L. plantarum* 13A, 41G, 38I and 41P and *P. acidilactici* 40J against a range of indicators. Presence of zone of inhibition was presented as ‘+’ and absence of zone of inhibition was presented as ‘−‘.

Indicators	13A	41G	38I	41P	40J
*L. cremoris* HP ^a^	+	+	+	+	−
*L. cremoris* MG1363 ^d^	+	+	+	+	−
*L. cremoris* NZ9000 ^d^	+	+	+	+	−
*L. paramesenteroides* NCDO869 ^b^	+	+	+	+	−
*L. mesenteroides* NCDO2028 ^b^	−	−	−	−	+
*K. aerogenes* NCIMB10102 ^c^	−	−	−	−	−
*E. faecium* NCIMB11508 ^c^	−	−	−	−	+
*P. aeruginosa* PA01 ^a^	−	−	−	−	−
*L. innocua* UCC3 ^d^	−	−	−	−	+
*L. innocua* DPC3565 ^d^	−	−	−	−	+
*L. innocua* DPC3566 ^d^	−	−	−	−	+
*L. innocua* DPC3567 ^d^	−	−	−	−	+
*S. aureus* NCDO949 ^b^	−	−	−	−	−

^a^ Strains obtained from American Type Culture Collection (ATCC); ^b^ strains obtained from the National Collection of Dairy Organism (NCDO); ^c^ strains obtained from the National Collection of Industrial and Marine Bacteria (NCIMB); ^d^ strains obtained from the University College Cork (UCC) culture collection.

**Table 3:** Antibiotics susceptibility of LAB. Results were expressed as R (resistant), MS (moderately susceptible) or S (susceptible).

	Penicillin	Oxacilin	Ampicillin	Vancomycin	Gentamicin	Tetracycline	Erythromycin	Streptomycin	Chloramphenicol	Mupirocin	Rifampicin	Nalidixic Acid
*L. curvatus* 40A	S	R	S	R	R	S	S	R	R	S	S	R
*L. curvatus* 41A	R	R	S	R	S	S	S	R	S	S	S	R
*L. curvatus* 15E	S	R	S	R	R	S	S	R	S	S	S	R
*L. curvatus* 15A	S	R	S	R	R	S	S	R	S	S	S	R
*L. sakei* 41D	MS	R	S	R	R	S	S	R	R	S	S	R
*L. sakei* 42C	S	R	S	R	R	S	S	R	S	S	S	R
*L. sakei* 38F	R	R	S	R	R	S	S	R	R	S	S	R
*L. sakei* 39F	R	R	S	R	R	S	S	R	R	S	S	R
*L. plantarum* 37I	R	R	S	R	R	S	R	R	S	S	S	R
*L. plantarum* 38 I	R	R	S	R	R	S	S	R	S	S	R	R
*L. plantarum* 13A	R	R	S	R	R	S	S	R	R	S	R	R
*L. plantarum* 37F	R	R	S	R	R	S	S	R	MS	S	S	R
*L. plantarum* 41O	R	R	S	R	R	S	R	R	R	S	S	R
*L. plantarum* 41G	R	R	S	R	R	S	S	R	S	S	S	R
*L. plantarum* 41E	R	R	S	R	R	S	R	R	MS	S	R	R
*L. plantarum* 41P	R	R	S	R	R	S	S	R	R	S	S	R
*P. acidilactici* 40J	S	R	S	R	R	S	S	R	MS	S	S	R
*P. acidilactici* 40I	S	R	S	R	R	S	R	R	R	S	S	R
*L. coryniformis* subsp. *torquens* 42L	S	R	S	R	S	S	S	MS	S	S	S	R
*L. coryniformis* subsp. *torquens* 42M	S	R	S	R	S	S	R	MS	S	S	S	R
*L. coryniformis* subsp. *torquens* 14I	S	R	S	R	S	S	S	R	S	S	S	R
*L. coryniformis* subsp. *torquens* 15I	S	R	S	R	S	R	R	R	S	S	S	R

**Table 4:** Hydrophobicity (%) and autoaggregation (%) of LAB isolates. Results presented as mean ± standard deviation (SD) of three independent experiments.

Strain	Hydrophobicity (%)	Autoaggregation (%)		
		3 h	5 h	24 h
*L. curvatus* 41A	8.2 ± 0.6	10.5 ± 0.4	13.9 ± 1.1	28.9 ± 1.5
*L. curvatus* 15E	7.8 ± 0.7	10.2 ± 0.9	12.3 ± 1.3	32.0 ± 6.2
*L. curvatus* 15A	18.1 ± 2.4	9.7 ± 2.9	15.1 ± 1.4	50.7 ± 5.8
*L. sakei* 42C	4.9 ± 0.5	12.6 ± 0.3	14.0 ± 0.4	45.1 ± 5.0
*L. plantarum* 38I	37.1 ± 0.3	11.4 ± 0.1	19.0 ± 1.0	66.9 ± 5.2
*L. plantarum* 13A	34.5 ± 0.4	10.6 ± 0.7	18.1 ± 0.4	56.9 ± 2.8
*L. plantarum* 37F	31.2 ± 0.5	11.8 ± 0.3	20.4 ± 0.9	63.6 ± 2.2
*L. plantarum* 41G	37.4 ± 0.7	11.9 ± 0.4	19.7 ± 0.7	67.6 ± 4.1
*L. plantarum* 41P	39.9 ± 2.0	11.4 ± 0.1	19.5 ± 0.4	69.0 ± 4.2
*P. acidilactici* 40J	11.5 ± 1.4	10.6 ± 0.7	14.4 ± 0.8	28.7 ± 3.8
*L. coryniformis* subsp. *torquens* 42L	38.8 ± 2.3	15.4 ± 1.1	22.5 ± 0.7	48.0 ± 3.6
*L.coryniformis* subsp. *torquens* 14I	42.2 ± 0.7	15.6 ± 1.3	22.5 ± 0.6	52.6 ± 4.5

**Table 5:** Survival of selected LAB isolates under simulated gastric juice conditions at 37 °C. Results presented as mean ± SD from three independent experiments. No survival was presented as ‘−‘.

Strain	Mean ± SD (log_10_ CFU/mL)			
	0 h	1 h	2 h	3 h
*L. curvatus* 41A	8.16 ± 0.17	−	−	−
*L. curvatus* 15E	7.62 ± 0.45	−	−	−
*L. curvatus* 15A	7.53 ± 0.59	−	−	−
*L. sakei* 42C	7.46 ± 0.23	−	−	−
*L. plantarum* 38I	9.45 ± 0.02	8.03 ± 0.41	3.96 ± 0.74	2.09 ± 0.13
*L. plantarum* 13A	9.40 ± 0.20	7.94 ± 0.43	4.32 ± 0.82	3.11 ± 0.97
*L. plantarum* 37F	9.57 ± 0.11	7.90 ± 0.62	4.41 ± 0.42	2.98 ± 0.35
*L. plantarum* 41G	9.42 ± 0.22	7.84 ± 0.43	3.59 ± 1.21	2.00 ± 1.41
*L. plantarum* 41P	9.34 ± 0.20	7.10 ± 1.17	5.78 ± 0.55	3.31 ± 0.93
*P. acidilactici* 40J	9.56 ± 0.06	5.57 ± 0.42	4.40 ± 0.38	3.99 ± 0.43
*L. coryniformis* subsp. *torquens* 42L	8.79 ± 0.24	2.69 ± 0.53	−	−
*L.coryniformis* subsp. *torquens* 14I	8.40 ± 0.44	2.91 ± 0.20	−	−

**Table 6:** Survival of selected LAB isolates in MRS broth supplemented with 0.3% bile salts at 37 °C. Results presented as mean ± SD from three independent experiments.

Strain	Mean ± SD (log_10_ CFU/mL)			
	0 h	1 h	2 h	3 h
*L. curvatus* 41A	7.59 ± 0.22	7.36 ± 0.11	7.08 ± 0.14	6.72 ± 0.18
*L. curvatus* 15E	7.57 ± 0.20	7.21 ± 0.23	7.07 ± 0.11	6.83 ± 0.34
*L. curvatus* 15A	7.22 ± 0.32	7.08 ± 0.23	6.90 ± 0.20	6.64 ± 0.21
*L. sakei* 42C	7.41 ± 0.11	7.06 ± 0.03	6.96 ± 0.23	6.52 ± 0.14
*L. plantarum* 38I	8.90 ± 0.18	8.77 ± 0.14	8.57 ± 0.20	8.41 ± 0.08
*L. plantarum* 13A	8.81 ± 0.03	8.86 ± 0.12	8.47 ± 0.14	8.22 ± 0.08
*L. plantarum* 37F	8.83 ± 0.15	8.70 ± 0.08	8.48 ± 0.16	8.23 ± 0.12
*L. plantarum* 41G	8.89 ± 0.15	8.81 ± 0.22	8.60 ± 0.10	8.38 ± 0.11
*L. plantarum* 41P	9.01 ± 0.08	8.61 ± 0.14*	8.55 ± 0.14	8.39 ± 0.09
*P. acidilactici* 40J	9.01 ± 0.12	8.92 ± 0.04	8.48 ± 0.06	8.25 ± 0.05
*L. coryniformis* subsp. *torquens* 42L	8.59 ± 0.13	8.20 ± 0.26	7.94 ± 0.26	7.78 ± 0.26
*L.coryniformis* subsp. *torquens* 14I	8.73 ± 0.05	8.33 ± 0.40	8.09 ± 0.15	7.91 ± 0.22

## Data Availability

Not applicable.

## Supplementary Materials

The following are available online at https://www.mdpi.com/article/10.3390/foods10071519/s1, Table S1: Standards for interpreting the inhibition zone diameters for antibiotics used in this study. Results were recorded as: R (resistant), MS (moderately susceptible) or S (susceptible), Table S2: The source of origin along with the incubation condition where the 22 LAB isolated from, Table S3: Average mean concentration of organic acid (mmol/L) and standard deviation (SD) of organic acids produced by LAB, obtained from three independent experiments, Table S4: Amino acid sequences that encode *bsh*, *mub* and *fbp* genes in probiotic candidate strains, Figure S1: (GTG)_5_-PCR DNA fingerprints of LAB isolates obtained from prosciutto and pancetta. Lane 1–8: *L. plantarum* strains 41G; 41E; 41O; 41P; 13A 37F; 38I; and 37I. Lane 9–12: *L. sakei* strains 41D; 42C; 38F; and 39F. Lane 13–16: *L. curvatus* strains 40A; 41A; 15A; and 15E. Lane 17–20: *L. coryniformis* subsp. *torquens* strains 42L; 42M; 14I; and 15I. Lane 21–22: *P. acidilactici* 40J and 40I. The first lane was GeneRuler DNA Ladder Mix (Thermo Fisher), Figure S2: Representative spot-on-lawn agar plate of LAB isolates against *L. innocua* UCC3 indicator. The zone of inhibition formed by *P. acidilactici* 40 J and 40I were defined, whereas the zone of inhibition produced by *L. plantarum* 41P, 41O, 38I and 37I and *L. coryniformis* subsp. *torquens* 42M, 42L, 14I and 15I were hazy or less defined, Figure S3: Schematic representation of the gene clusters involved in the production of previously studied strains of LAB (*L. plantarum* NI326 and *P. acidilactici* K10) with bacteriocin producing strains isolated in this study (*L. plantarum* 41G, 38I and 13A and *P. acidilactici* 40J).
