# Supplementary material for: Lactic Acid Bacteria Diversity and Characterization of Probiotic Candidates in Fermented Meats
Source: Foods. 2021 Jul 1;10(7):1519. doi: 10.3390/foods10071519 (PMC8305854; doi:10.3390/foods10071519)
Supplement: Supplementary file 1 [file foods-10-01519-s001.zip › foods-1252670-supplementary.pdf]

Supplementary Material

Table S1. Standards for interpreting the inhibition zone diameters for antibiotics used in this study. Results were recorded as: R (resistant), MS (moderately susceptible), or S (susceptible).

Antibiotic	Mechanism of action	Concentration (µg/disc)	Zone of inhibition			Reference
			R	MS	S	
Penicillin	Inhibitor of cell wall synthesis	10	≤ 19	20 – 27	≥ 28	Charteris et al. [1]
Oxacillin		5	≤ 14	15 – 19	≥ 20	Sharma et al. [2]
Ampicillin		10	≤ 12	13 – 15	≥ 16	Charteris et al. [1]
Vancomycin		30	≤ 14	15 – 16	≥ 17	
Gentamicin	Inhibitor of protein synthesis	10	≤ 12	–	≥ 13	
Tetracycline		30	≤ 14	15 – 18	≥ 19	
Erythromycin		15	≤ 13	14 – 17	≥ 18	
Streptomycin		10	≤ 11	12 – 14	≥ 15	
Chloramphenicol		10	≤ 14	15 – 19	≥ 20	Sharma et al. [2]
Mupirocin		200	≤ 14	15 – 19	≥ 20	
Rifampicin	Inhibitor of nucleic acid synthesis	30	≤ 14	15 – 19	≥ 20	
Nalidixic acid		30	≤ 13	14 – 17	≥ 18	Charteris et al. [1]

Table S2. The source of origin along with the incubation condition where the 22 LAB isolated from.

Name	Growth condition	Source
L. plantarum 41G	30 °C	Prosciutto
L. plantarum 41E		
L. sakei 41D		
L. sakei 42C		
L. curvatus 40A		
L. curvatus 41A		
L. plantarum 41O	37 °C	
L. plantarum 41P		
P. acidilactici 40J		
P. acidilactici 40I		
L. coryniformis subsp. *torquens* 42L		
L. coryniformis subsp. *torquens* 42M		
L. plantarum 13A	30 °C	Pancetta
L. plantarum 37F		
L. sakei 38F		
L. sakei 39F		
L. curvatus 15A		
L. curvatus 15E		
L. plantarum 38I	37 °C	
L. plantarum 37I		
L. coryniformis subsp. *torquens* 14I		
L. coryniformis subsp. *torquens* 15I		

Table S3. Average mean concentration of organic acid (mmol/L) and standard deviation (SD) of organic acids produced by LAB, obtained from three independent experiments.

	**Lactate**		**Acetate**		**Propionate**	
	Mean	SD	Mean	SD	Mean	SD
L. plantarum 41G	117.13	0.53	41.25	0.61	3.92	0.16
L. plantarum 41E	114.99	0.09	41.49	0.06	3.65	0.03
L. plantarum 41O	158.02	0.22	46.77	0.07	3.61	0.03
L. plantarum 41P	157.09	0.29	46.78	0.03	3.43	0.05
L. plantarum 13A	131.20	0.17	43.82	0.03	4.90	0.04
L. plantarum 37F	106.67	0.14	40.94	0.05	3.26	0.02
L. plantarum 38I	133.13	0.13	45.16	0.07	2.81	0.01
L. plantarum 37I	138.91	0.42	45.84	0.04	2.97	0.04
L. sakei 41D	80.19	1.95	37.74	0.20	0.00	0.00
L. sakei 42C	68.75	0.08	37.68	0.04	0.00	0.00
L. sakei 38F	81.90	0.02	37.65	0.05	0.00	0.00
L. sakei 39F	81.60	0.20	37.81	0.08	0.00	0.00
L. curvatus 40A	71.14	0.02	37.03	0.03	0.00	0.00
L. curvatus 41A	74.82	0.05	36.86	0.07	0.00	0.00
L. curvatus 15A	78.07	0.10	36.86	0.32	0.00	0.00
L. curvatus 15E	75.79	0.23	36.50	0.31	0.00	0.00
L. coryniformis subsp. *torquens* 42L	86.87	0.05	37.56	0.21	0.00	0.00
L. coryniformis subsp. *torquens* 42M	79.37	0.01	37.35	0.04	0.00	0.00
L. coryniformis subsp. *torquens* 14I	89.89	0.18	37.43	0.03	0.00	0.00
L. coryniformis subsp. *torquens* 15I	88.54	0.18	37.33	0.14	0.00	0.00
P. acidilactici 40J	88.08	0.05	38.40	0.02	0.00	0.00
P. acidilactici 40I	88.53	0.06	38.35	0.05	0.00	0.00

Table S4. Amino acid sequences that encode *bsh, mub* and *fbp* genes in probiotic candidate strains.

**Isolate**	**Gene**	**Amino acid sequence**
L. plantarum 38I,<br>*L. plantarum* 41G	*bsh*	MCTSLTYLDTDNHRYFARTMDFPTTTPWRPIFLPRRYPWPTGLATTRMTQYAILGGG<br>RLPDHFKACLMADGINEAGLMCAELYLPHAVEYATQPQVNQINLTPQAFINWALGE<br>HQSVAAVIADLPSVNLVGASWGDDTGEVYPFHWYLSDAHTSVVIEPTGGPLTAQPN<br>PAGVLTNTPVLSDHQRRLNLYLAISGNQITTATRQAAQHVIQTKQPLPSGPIPTDRFIH<br>MALRRLGTPQLAPQQVPTTLFRWLQEVSLPYHADRRHLISHNYTHYRCLITLATRTY<br>RFIPRTTGHEQRLTLTPEMAATWRTPYLFPAD
	bsh	MCTSLTIQTTAGDQFLARTMDFAFELGGRPVAIPRNHHFDSVTNADGFDSPYSFVGT<br>GRDLNGYIFVDGVNEHGVSAAALYFSGQAHFTQQTKAGKVNLAPHEVLMWILGNV<br>KSTAELGERIADLNVMEAAAPLLNIVVPLHWIISDKSGSTYVLELENDGVHYMKNPV<br>GVMTNTPDFEWHLKNLSNYVNLQPGPHPSRQYGDMTVNPFGPGTGALGMPGDYTS<br>VARFVRTVFMREHTDAVTTDAEAVNALSHMLNSVEIPKGVKMQDNGTPDYTQYRA<br>YMSMNEPAFYMQPYADQTITRVELTPALMTAAQPTEFELKTTQQFRLAN
	bsh	MCTAITYQSYNNYFGRNFDYEISYNEMVTITPRKYPLVFRKVENLDHHYAIIGITADV<br>ESYPLYYDAMNEKGLCIAGLNFAGYADYKKYDADKVNITPFELIPWLLGQFSSVREV<br>KKNIQKLNLVNINFSEQLPLSPLHWLVADKQESIVIESVKEGLKIYDNPVGVLTNNPN<br>FDYQLFNLNNYRALSNSTPQNSFSEKVDLDSYSRGMGGLGLPGDLSSMSRFVRAAFT<br>KLNSLPMQTESGSVSQFFHILGSVEQQKGLCEVTDGKYEYTIYSSCCDMNKGVYYYR<br>TYDNSQINSVNLNHEHLDTTELISYPLRSEAQYYAVN
	bsh	MCTSLTYTNSHGGHFLARTMDFNVDFETRIMFMPRHYRVTGDLGDFTTTYGFIGAG<br>RQLNHEIFTDGVNECGVSIAALYFPNHAIYQPHSNQDKIDLAPHDFVAWVLGKITSV<br>ADLRERVKDVQLISSTAELINEIPPLHFIISDQTGETAVLEPTSGELRLLNNPVGVLTNS<br>PNLKWQLQNLSKYGTLTNTERPLNKFINYQPGSQGPGTGALGLPGDYTSMSRFARTV<br>FLKHYAQVPATTTDTVNLLQHILNAVTIPKGAKVAANGQATYTEYRSYMDLNHQTY<br>ALELYENPGVIQQVNLTDHLLEKQTVPLEYALSRTPHVQLLTPDIATLPVAH
	mub	LKPNNVNNQNKRHQSRWVITSATAMILTTLTIASQAAAADDTVTTTTNEPTNSQLNT<br>NTQVNATQVNLKADTSTSVSTIKSDQSAVAATSPTTSTGSPSEHSSSVNTNPQQQSAN<br>PASQSQATTTSESTPTTDIKHPTQTAPAQTASASTTEPTTESNTESATDSQAKATTTDN<br>QASKQPSQQAVPASSNSTTTEVNTQSATSSASTDDKIVTNVNQEKLVLKTNQPVVRA<br>ISRTASENINDWMPNTLLQQEVLSQLRKQNSDRTWNSAADITKADMLLLTTYYGKD<br>TYIDGKTSYSLEGLQYATNLTTVWLNNNLNAPSGSYYSDVTDISPLANLQKLQVVNI<br>QQNRIMDISPLANLKNLTEVDAAYNHISDFSPLKGFKNLKGTFSNQFITLPPAYISADN<br>NIATLAIDCYLPDGSKVQLKPNNGVGETVFYKNGQLYVRWYFNGAGGGNYDSNGHI<br>YYTNMKPQQPGLTGPTFNGTTVIPMDDYYFMTAASDGNNFVVVRPYVLAATAAPIT<br>VKYVDALTGESLVTTDLTLNGIVGQPYTTQRIDDELPNYDFTNIVGNASGVFTADAQ<br>TVTYYYTRKDAGDITIHMVDTNGNLVYEPQILPGKHNLGNAYNLDAPTFDHFKLQQ<br>TIGNAAGVFTTDPQSITFVYVRLDAGNITVKYQDKQGHQLKPDKTVSGSQSLGQTYT<br>TEPLGIENYTLMTTPANATGTFTDQEQTVIYVYVRRDAGQIVVKYQDSAGNPLAPDK<br>LLDGKEQLGTAYQTAAISIPNFYLVATPANATGTFSTDTQTVIYQYTRSNAGHITVKY<br>QDANGTTLAPDDILTGNGQLGRPYQTNAKTIENYRLFQTPANATGQFSDQAQTVIYV<br>YTREDAGDITVQYLDENGQQLAADNVLSGQGQLGQPYETSPLNINGYTVKSTQGNT<br>TGTYTAQPQRVVYIYERTAGQPVTAKYQDQDGKSIHPDVVHSGYLGDNYSTEQLAI<br>DGYTFKTVQGDVNGTFGTTAKTVTYVYERTAGLPVTVKYLDEHGKSIHPDVVLSGY<br>LGDSYSTKQLVIDGYTFKAVQGDVSGTFGTTAKTVTYVYTENTPTIPDTQGTVTV<br>HYVTKDGIKLNEPTVLSGKTGTTYQTVPLTFTDHELVGQPENATGLFTADNVDVTY<br>VYQATDTAGTDDIIDPEEPEQPTKPVEPTTPETPNEPGTTVTQPDRIKPTQPAVAVKPA<br>ATVKPALKPAAAQASLVKTTSPVTEHSAQLPQTDEQTGKLAVILGLLLSVVTFGFYG<br>KHRQS
	mub	VSFLDRLKGMLQALNSTEAATSATEAPRSIAAQTAAAPTVNQTEALVLVHHLDQDG<br>NELQAADMIAGTIGEEIHLPAVSITGYHLVHIEGLTRWFTTPQASITLTYERQAGQPV<br>WMYAYDIDRRELIGRPTMYRGKLGTPYEVSAPTVAGFKLLRSVGDVTGEYTTTSKT

VLFFYRNQNWQQTDLSTGFVQVNKLTAVYPYPGATTTNYLTKLQPGSTYKTYMRV
RLVTHETWYAIGDDQWIPETHLQLTTGDTLLLKLPAGYRVQNKRPVRQTGVVSFVP
GKQVHTYIEPYGRYLTTVTHGDTVNLIERMADDNGVVWYRLQDQGYLPGRYLTKL
DPPFA

mub MSKDNQKVTGDSIYRVKMYKDGQRWVCAGATTLALAAGLVFANVNASADTTPAN
ETKTEQVTSGASSSATSQAATSDASSASSTSQATSTTSQSTSTVSAATQSATSTTSAAS
SVASQNDTTQTAVATSAKVQSRAASVDTTATVQQATPTVTAPTSSATPKVTSEATLT
TSEAASGSTSEATSTVATGSGSLAASHDVNANNVKVDGTTVAKTVKGQTYDMQVV
LTKNKAIDWNTGRTNGQFSIAPAGSSETANGTWKAVSYQINQNTPVTIGTGSTIVVPV
KDVINATTLTFNYGYTATNDTNSNSDYLSINDIPNADDKAHIEAAGNTVLYQNGWD
YTAQTTTDVTTTTPAEDAQDVSVAYVVITGFNADGTPKYTQIAGGVTKSGKVGTTFT
ISPATIDGYALYGASNNVKPGDNGLTGTVDSVATNNEIVLVYAENTGIVANYQTADG
TTLATPNQYTQGTDKTFTRAGGTYALTAPSLDGYTYVGYKIGDGDVQSGNVAAGTL
VAGANTVTFVYAPVVEQSDVTVNYVDESGNTIKAATTQTLDNGSTYTVATPTIDGY
TYKSADGALTGMVDGNKTITLTYTKNAAPVEQSTITVNYVDADGKTIKTATTQTLD
NGSTYKVETPTIDGYTYKSADAALTGTVDGNKTITLTYTKNAAPVEQSTITVNYVDA
DGKTIKAATTQTLDNGSTYKVETPTIDGYTYKSADAALTGTVDGNKTITLTYTKDST
TPVENKANLTINYVDADGNTIKASNVTEYIVGQAYTVGQPEIAGYTYDHATGDAIAG
TIAYKGNTVTLVYTKNGGTTPTEQTKTITVNYVDADGNTIKSATTTTYKVGDTYTVA
TPTIDGYTYKSADGALSGTVADDATITLTYAKNDNGGSTTAPTTAPGTGDNGNNGG
GTTTTAPTTAPGTGDNVNGGGTGTTTTAPVTTPSDDTVDNGNGSSNNGSSTTTTSTA
PATTVSDDEVTPTTTATTNNGTSGVVPASASLKPVVTTKTTTSDAKTLPQTDEDENG
TALAVLGLSTLLMGSALYFGVSRRKHEA

mub MTNYSYLFANLLNVTAIDGLANLNLTGVTDISWLFLNCSQLGALDLNSWDVSSVIR
MEGTFQNCTKLVTLNVANWNTDSLQYLIDTFNGDSSLTSLPVGKWNTSKVATMMR
TFTDCSSLTSLDIANWDTRVVTNMSAIFRGMSKVKSLPIDKWQTGRVVNMQLVFSG
DTSLESINVANWDTSRATALDGTFAKLPNIKSLPLDNWNTSNVQTIRSTFYGDTNLTQ
LPIDNWNVGKVFDFNSTFSGCASLTTAPVANWNTQSATNLGYTFEGMTSLTSLPVDN
WQTGTVTNMAGTFSRVSQLKSLPISKWNTKNVQNMAGTFSEMSSVTALPVDNWQT
GNVTTMRGIFTKVSQVKNLPVGKWDTAKVVDMGQVFYGNPQLTSLPIENWNTSSA
TDFSQLFAEDSGLQTLSLGAWNTTKVTNFESVFQNTSLDKLDLTGWNTNSAQTYTN
AFSSKLPPKRLLLGPSFNFFKSESWHLPNPSSEAPYIGKWRSLNNKKVYTSADLMTKY
DGKTIVGEFEWATGNTITVKYVDAAGKSLAPDTKISGATGDAYHIKPIEIEGYVPDQP
DGVQGNFTDKDETITLMYSPGGIMFASAPQTINFGQNPITGKSESYGASYDTGLVIQD
GRSIGSTWSLNATLSASGFTSKQSARPLAAVLSYKDQQTGGESILTPGVARLIVNNHQ
TVSNQGVNILGQKTALGALSLQVPTDRALTDTYQATVTWTLNQGVPNR

mub LNKRKIITNNPPKWHLITGIAATILAGIILTNQDAFAATDSPITPTTTAPTVQQTAPTNP
LSGSQVTLTSTTGSSATGSTTTSSPAATSTAALPAKSTATSGSLMSAMASSSATSGHA
AEPSSSVTEAASTNNLIPTSAAMASSATTKYPTDTTATPNASSSPTSAESSTPNKAMST
SQQTDSSSVIHSTTPASSTSMPVPTSVGSMTTAKTASSAAPDVTNSTAANSTAPTSVT
TTDSAAESMPLSTSSETSSEKSAAASTTSTSQISDGSEVIHPMTSAISSSSSAPTSGAKM
AASAASAASASVITSAVNSIAASTYSADASAASVESAATPDTSHATVPASTATSAATT
FQITSVINSLASSTYSEYAEQANAEAASAATTAEKPATSVGTVVPTAATTPIESIDTW
MPNKHLQEAVLRELQALKLPDHQFKSVNDITKDDMQLLTQFYGENTYIDGHTPYSL
EGLQYATNLKTIWLNNGLNALGGYYNGDVTDISPLAGLTKLTVLNIQHNRVSDLSPI
THLTNLQELDVAYNHIADLSVFKDLPNLKTTTYLGQTILEPLVYVDQDTTSATLKNR
FYLPNGQQAVLKSQAAILKPVQLTPNGQFYYRFYFNGAGKAVNGDLSNVVPDGQG
GLTFNQLVPQIPGFTGDANGQFVTNGVSINVVPNDKNFYLVAQGSDGSSPVFHVFQP
YVLAAKAAPVTIHHVDRNGAALRDSEELTGLVGEDYQSTPADITNYTHVETQGAPQ
GTFSAEPQAVTYVYDKTAGAPVTVSYQDEQGKTLQPDTTCNGLAGDPYTTKPLEIA
GYDLTKTPDNAAGTFTAEPQHVIYIYTKQVPQPVTASYQDEDGKALQPDITHTGEIG
AAYETKALEIPDYDLVKTIGNATGTFTKEPQQVTYIYTKQIPQPVTASYQDEDGKTLQ
PDITHTGEIGAAYETKALEIPGYQLIKTPTNATGSFTKEPQHVLYVYEKQAVLPVTVS

		YQDADGKPLRADIVLSGDFGQNYQTEQLSIPGYVFNKVVGPTIGTFGTTAQHVVYTY<br>TPESSEPEQPTPGPAPQPVPEPQPTPAPQPEPTPQPSPTPQPSPAPQPNPAPQPSPAPQPN<br>PAPQPGSSSLAKAPVSQGTTTSQSSPTTSPQPTPIAPVSALAQPGKQQAPATAATHNL<br>GQLPQTSEQSEHVATLGGILAALFTGLGWLGLAAKFKKRE
	fbp	MSFDGLFTHAMVTELRQTLVGGRISKINQPYQNELILTIRANRKNHPVLLSADPTYPR<br>IQTTQIPYVNPAVPTNFAMMMRKYLQGAIVTDVSQVANDRVVHLTVTTRNELGDAE<br>TLTLIIEIMARHSNVILVDNQTGKIIDVIKHVGADQNRYRLLLPGATYIEPPKQDKQDP<br>FTPNTDFHTLVTDYPNEDVLAKQLQQHYQGFGRDSAQQLAADLHQPGNLDHHYQA<br>FLAQFDQPQATLITLPNNKTMFAAGLFTHFGKATRQFDSLSSLLDFYYADAAQRERV<br>QQQAGNLIRVVKNNLKKNRNKLKKLEKTLANTKQADELRLKGEILTTYLHEVKRG<br>MTEITLPDYYHDNAPLKIQLSNQLSPSRNAQKYFSRYQKQKNAVGFVGEQISLTQAEI<br>DYLDNIQTQIELASPADITEIREELTQQGYLKQHKTKKKQRSSRKPSQPQEFTASDGTP<br>IFVGKNNRQNDQLTLKTARKSDYWLHTQKIPGSHVIIHSDDPSDQTLTEAANLAAYF<br>SKARDSATVPVDYVQVRRIRKPNGAKPGFVIYEGQKTLYVSPNAELVEQLTPHA
	fbp	MTTTTDVDLVQTGLAHLIHHTRKQIESLTKALDHLPDVSDLQLKGTLLKTYASQIEG<br>HHHFVELPDYQGHQLSIKLDIKKSIIENAEDYFHRYHKSKRGQATVQQNLATAKTEL<br>YQQLATQAAFDPNDPQAVAALKQTLIAAGAIPTHVLHSSKAPTPAHPRRFYTHDHVL<br>VEVGKNSRQNDHLTLTARKDYYWMHAGGEIPGSHVVIHSNHPSEQTLQEAAVLTAY<br>YSKGRQMNRVPVDVLTVGQMRKPKGAKAGLVTFSGPARTITVVPDATLAADLRDQ<br>EDIHHDAD
P. acidilactici 40J	*bsh*	MCTSIELTAENGAKFWGRTMDLAMTMFGEDGGAESVITTIPAEAKIASQLTDWTAK<br>YATMGVGVKGTPILFDGINEAGLAGDLQVLFESTADSLENLKQRGLTPLMNTEFVTY<br>VLTHFKSVAEIREHYQELGLADQATQVNGQGFTFPLHYNFVDESGDGVVLEPVENG<br>AFKLYDSVGVVTNSPEYSWHTTNLRNYLGLTDVDVKDPRNYKNGVTLPPIEGGTGY<br>GMAGLPGSYTSPARFVRSFTIANAMDDFAADRGIAQLYAAFRPVIIPEGIERKTADAPI<br>SDYTRYWSGYDLRKRAVYVQTGLGLAFTKQTLNADAQEISYTTIDRGDYVHEV
	mub	LGTNHYQRFYNYGRKWFITSLVGVTIGLNVLLSPISVAASDQVSKSTYPASSPQIAPQ<br>EAASRPPRLDYWGSSASAIASSSADPHHYFWGNKHHHHHWHHHCHATVSVRYVDQ<br>SGHLLGTGQAYCPHGPYVGEPYVSQPRSIKNYRLVGTAPDSLPTRGRLNKDGDNGM<br>VTYIYAPIYHFKAKTINETIHYVDKNGQTVAPTHVAQPITFVTVDNLAEHVTKNYFSN<br>TSTDFQMDDQGNPRDSENWHSGTQATFTEVPDPLPHGYQLTDPAKDAVPATPVTPA<br>STDLNITVRYQLKKKTAVIRWIDDRTGKIVSSKTVNVDVKHPQANLTETFDHQDYRL<br>VSSDLPQGSLTFDEIKESGHLYEVHLRSQTPPVLPAEVKPTPSEMSSSSQPVTPPALAP<br>AEQRPALPVSPLQRATPAIQPAVAGRHPQQLSKSLSSSSSRPQSAQSQPRYPVPTGTIP<br>LAVNDQQPAPVPDLLARLNLKWYFPRDHHPVLAFDDWNNHVQKIIKLNGDTKSHT<br>QLGQVWYKMSGRLVMGQRVRKSPGSL
	mub	MFDSCANLKNLDLSHWNTSNVTNMNYMFEACGVSRLDLSHIDMSNVTRYDRMLTA<br>MSNLRVLVLGKKTKIAGAHLSEPSFIYTFNRWVAVAGGTEDNPLGKQQYTSHELMD<br>LYNPSMADTYVIRPFKTINEDQDVTQTIHYVNKNGGQMLPDHTETRRYSRKGHQNP<br>DTGEIYWEPWKIADGENTYFDPVYSPKISGYTPDIRVVDKTIIDDELREKGGIEVTVTY<br>SPNALTGSVTYWDDEDNKKVSTGSLNGSTDEDGNFVINVPAHYEIDPENNPNGYVD<br>GQTVHFTWSAKDGANDFTVYLRHKHRKVDPDDPEWSKETTWTVNETVHYVDNQG<br>KTVYPDTSAMLTYHREVVIDEVNNSVISRGEWQPDTSNKFAGFDTPIVDGYVADHA<br>HIEAQTVAKPGKPNQDITLTVVYYPSDVDVDPEYPIVPGTPVDPVNPDSPEYPAGMD<br>VNDLNKDLTETIHYVDKDGKQVAPDRTITVHYTRKGHVHFKQDGTATISYDPWEAD<br>EDYPAVDSPVIDGMFPDKQTVAEVDGNSKDDDFEVKVTYYPSDVDVDPDNPIAPDT<br>PVDPSNPDSPDYPAGVDVNDLNKDLTETIHYVDKVGNQVAPDQKVTVHYTRKAHV<br>HFNQDGTAEVTYDPWVADGNYPSVDSPVVDGMVPDTPTVKEVDGDSVSADFEVTV<br>IYHGSDIDVDPDHPIAPDTPVDPSDPDSPSYPSGVDVDDLNKDVTETIHYVDKDGKQV<br>APDKTVTVHYTRKGHVHFNQDGTAEVTYDPWKADSDYPAVDSPVVDGMVPDTPT<br>VKEVDGDSVNADFEVTVIYHGSDIDVDPSDPVSPDTPVDPDDPNSPDYPAGVDVNDL<br>NKDVTETIHYVDKNGKQVAPDKTVTVHYTRKGHVHFNQDGSAEVTYDPWKADGD

		YPAVDSPVVNGYEPDQPEVPAMDANSVGNDFVVNVIYHVTDDDHQVTPPDDDHDN<br>PTPDHPETPDDNNNDSQVPVDPETPDDDNNNQVPANPEVPNGDNGNSVPNQPEIPNG<br>DHNENQGSEDPVLPADNSGEVDGTAATPDTSNEYNKSDQFPGEPVSAVRTNEDNQIS<br>AKERTTLVKRGRLPQTNEVESNIMSEVGVLSLLTILLGFFGWGRKRKRDGEE
	fbp	MAFDGLVTHAMVNELRSLESGRVAKIYQPYQSELVLTIRANRKNVPLLISSHPNYAR<br>VQVTNQALSNPATPSNFVMSLRKHLEGAILKSVKQLNNDRIINFYFSHRNDIGDEEDV<br>ILSVEIMGRRSNVILYLGSSQKIIDTIKHISADQNRYRMLVPGAQYITPPAQELLNPFTD<br>SNQQTWDNLLRDYPNYEVLAKQIQATFQGFGKENALELAYRLINGQTPATVVHEFL<br>DCFNASPKGFIYESQAKKLTYSAIQPTLTADQPNLQTFNSLSEMLDQYYFEKVQRDR<br>VQQRGHQLIRVVRNELKKNRKKLKKLQNTMAQTQYADSYRIKGEVLTTYLSQIKRG<br>MTSVELPNFYDQNQPITIKLSNQLSPSQNAQKYFKKYQKEKNAVKYVSEQIAHTESEI<br>AFLDNIETQIDLAKPEDLAEIKIELENEGYLRKQRNPKKRVKNVISKPEQFISNDQTPIL<br>VGKNDAQNDRLTLKTAKKDYYWLHAKDIPGSHVIVESSQPSEQTLIEAATIAAYYSK<br>GKNSANVPVDYVPVKNIRKPNGAKPGFVIYEGQRTLKVTPQITVVQKLMQKK
L. coryniformis subps. *torquens* 42L,<br><br>*L. coryniformis* subps. *torquens* 14I	*bsh*	MTGQSTTWRSNYATLGVGLTNSLVLYDGINECGLAGDLQVLMECGRASADDLEQR<br>GLQPILGEELVTYILTQCKDVAAVKELAATLALVDQPYSFMGQTAQIPAHYTFIDPTG<br>AMVVLESTDNGTFKLYDSVGVLTNSPEYDYHTTNLRNYISLDNLNKAKKTVGADLE<br>LEPIENGTGHGFFGMPGDYTSPSRFVRATLIAKNIDPFASSAGIPMLYNTFSSVMIPKG<br>LGRTPQHETVTDYTQYWSGYDLTARKLYVQDYACPTFTSKALDQTTDTITYTPIDLT<br>FQTNEI
	mub	VNFLKRLKAFFNPQQQVATAQLPMGPDYQPIARRRPILLPSPTRQAYLPISSHHQPSIH<br>VQPDSFVIVFYFTETGERLAKPQIISGVRGQAFKFTVHQFDDYYISRIENYHGYFVYPR<br>AIIQLIYAQQPAAPVIVFHFDENHHLLTPPEYLVGQLGQHYETHFLDSQLYRVQHVTT<br>NQVGHFSEQTQIVTYSYRPRVIRWANRYLTGFVRLTMPVTSYREPGKAALAQQLPTN<br>TIWRVYQKVQTTDRQTWYDLGGQWIATDHTERVEYYQQPKQQVIAAPLFQQAAVIP<br>GSRRAVVDFIPQRSLRTWTQPYGDAANYLQHGQIVNIIHGLILSNNSVWYELEDHTW<br>LEEHYLRLLSAGHDFTSPIKRLQ
	mub	VQLKTLEINGFKSFADKTVIQFDHGITGIVGPNGSGKSNITEAIRWALGEQSAKSLRG<br>GKMPDIIFAGSSERKPLNRAEVTLTFDNSDHQLKSDYDEVSVTRILYRDGTSAFYLNQ<br>KSCRLKDIVNLFMDSGLGRESFSIISQGKIAAIFNSKPEDRRGIIEEAAGVVKYKQRKK<br>EADSQLSATADNLHRVSDIIAELAQQVEPLKEQSSLAKDYLQQKKQLDQITKTLLVR<br>QIESRAAAKKEQQQALKTAQENVILARRNQTDFQTKVTAQKEQQQKNTQRLDILQQ<br>DQLRITKQQAELSGQAELAGERRRVAQNKLADLTAELKTQQQQLAELQQVLTTEKT<br>ALQQADADLKQKQQLVQNYTQAQQVDEATIKRKVEALRQQYIDQLQQQTTLHNEA<br>AYLAREQQQTKVRDEKQQAEQAALAQQTAELAAQVKTAEKTATQLKKQRTQQQQ<br>QQQQLTTQVQHDQQQYDQQQQQWYQALAIMQKVEAKQASLAEMKEDYSGFYAG<br>VRSILQHKTQLTGVIGAVAELLTVPAKYNQALEAILGGQLQAVVVSDEAAGKRSINY<br>LKQQHLGRATFLPLSVMQPQQLNSTQMQQIAQQPGFIGVAADLVKFAPEHANVMRH<br>LLGRIVIATDLTAAIKLAALLQHRVRIVTLEGDILNPGGAMTGGGRTKQNGLLAREQ<br>EQRDLTAQLAKMKQQLATKEQALQALKAQIEAANQQLSTTDEQLQQLARQELENQ<br>AILKDLTRQQAQLVRQQQAVAYTSQQQNDATADQQQKYLANQQQAKKVAATIEKI<br>QADLAAQQAALASWQENQAATASQRQQAQTDLAVAQEQQKQRQQTNREHVEQQS<br>ILQAAMVRNQQQCDQLTQRLNTQTKDHASNAADLKRAAEQLAQIDVQIKTVKEQR<br>EVLAATLQQQEQQLAHAQAVYQQQTAAQQRIEATLASLNDSLNHALTQLESDYNLS<br>FEAAKQAALPTDVAELQQQQKLLKLGLADLGEVNIGAIAEYERVSQRYEFLLAQRE<br>DLLSAREQLTTTMTEMDQEVSQRFKATFDATAKAFATIFPQMFGGGQASLSLTDPAN<br>LLTTGIEITAQPPGKKLQQLSLLSGGERALTAITLLFAIIHVRPVPLCILDEVEAALDEA<br>NVERFGRFLQRYDQQTQFVVITHRKGTMMAMDVLFGVAMQESGVSHMVSVALED<br>VKTEAHA
	fbp	MSFDGVFTHTMVNELSPLLTGARLSRISQPYPNELIITVRANRHNYPLLLSAHPSYAR<br>LQITEIPFVNPEKPTNFTMTLRKYLDGAILKSIKQIDNDRVVHLTFTARNEIGDQESLV

LIIEMMGRHSNIVLVNQADQRIIDTIRHVAHDQNRYRLLLPGATYIAPPQQNEADPFT
ATEPLYRTMPATPDIFAQAKALQQTYQGFGFDTALELATRLAQATDQDQAWHDFFQ
TLDQAPQPTITHDPTKKQLQFSPCAFISQHGTRTTYASLSELLDHFYRDKAQLDRVHQ
QGSDLIRLVQNELKKNKKKYRKLEQTLAASEKADTYRIKGEILTTYLHKVERGMTEV
TLPNFYADEAPIKISLSNQLSPSKNAQKYFTKYQKLKNSVHYVNEQLAATQQEIDYF
QGILTQIDLAAPKDLQDIQAELRQQGYLRTKSKQQKQRPKLSQPETFYASDGTKILVG
KNNLQNDRLTLKTARKTDIWLHAKDIPGSHVIITSAEPSEQTLVEAANLAAYYSKAR
LSATVPVDTVAVKKIHKPNGAKPGFVIYTGQKTITVTPDEQLVQKLSQQP

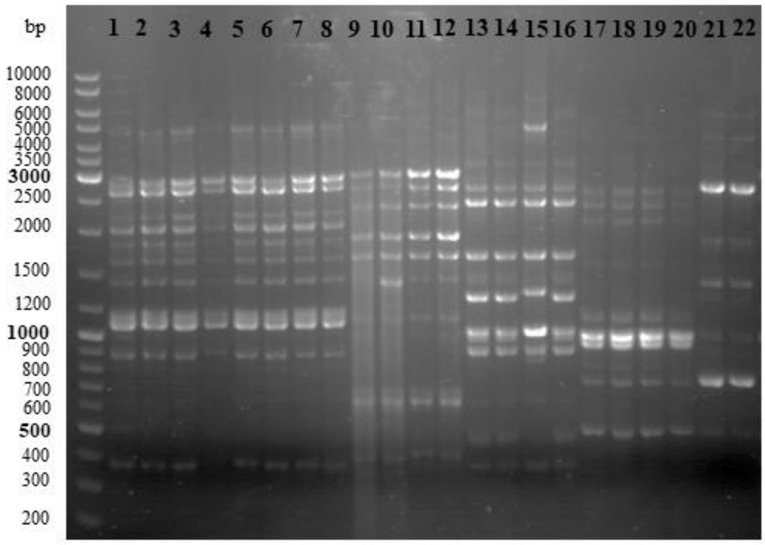

Figure S1. (GTG)$_5$-PCR DNA fingerprints of LAB isolates obtained from prosciutto and pancetta. Lane 1-8: *L. plantarum* strains 41G; 41E; 41O; 41P; 13A 37F; 38I; and 37I. Lane 9-12: *L. sakei* strains 41D; 42C; 38F; and 39F. Lane 13-16: *L. curvatus* strains 40A; 41A; 15A; and 15E. Lane 17-20: *L. coryniformis* subsp. *torquens* strains 42L; 42M; 14I; and 15I. Lane 21-22: *P. acidilactici* 40J and 40I. The first lane was GeneRuler DNA Ladder Mix (Thermo Fisher).

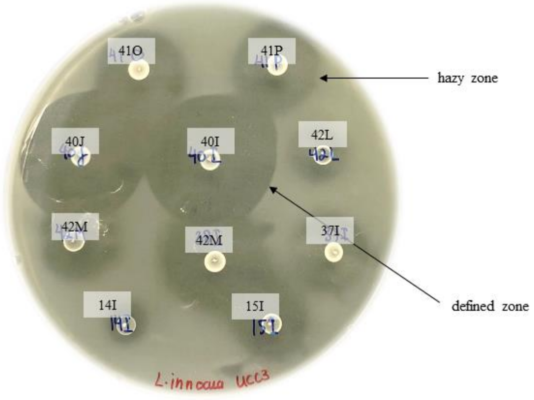

Figure S2. Representative spot-on-lawn agar plate of LAB isolates against *L. innocua* UCC3 indicator. The zone of inhibition formed by *P. acidilactici* 40 J and 40I were defined, whereas the zone of inhibition produced by *L. plantarum* 41P, 41O, 38I, 37I, *L. coryniformis* subsp. *torquens* 42M, 42L, 14I, 15I were hazy or less defined.

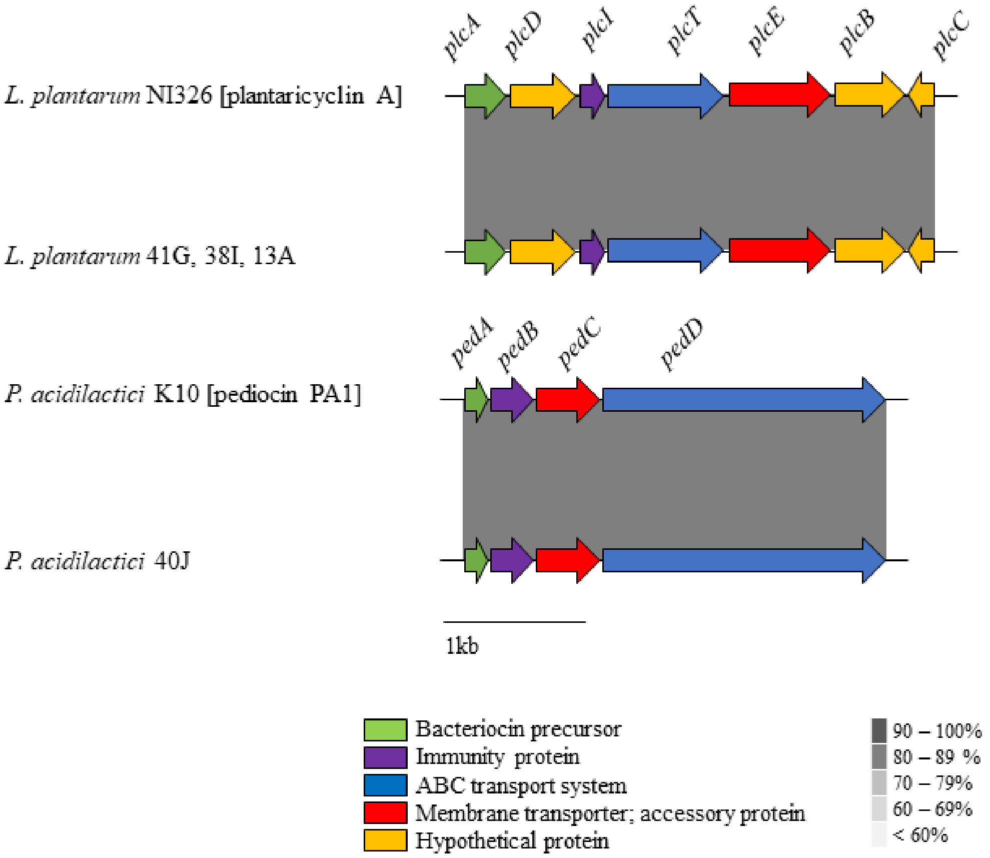

Figure S3. Schematic representation of the gene clusters involved in the production of previously studied strains of LAB (*L. plantarum* NI326, *P. acidilactici* K10) with bacteriocin producing strains isolated in this study (*L. plantarum* 41G, 38I, 13A, *P. acidilactici* 40J).

Reference

1. Charteris, W.P.; Kelly, P.M.; Morelli, L.; Collins, J.K. Antibiotic susceptibility of potentially probiotic *Lactobacillus* species. *J Food Prot* **1998**, *61*, 1636-1643, doi:10.4315/0362-028x-61.12.1636.
2. Sharma, C.; Gulati, S.; Thakur, N.; Singh, B.P.; Gupta, S.; Kaur, S.; Mishra, S.K.; Puniya, A.K.; Gill, J.P.S.; Panwar, H. Antibiotic sensitivity pattern of indigenous lactobacilli isolated from curd and human milk samples. *3 Biotech* **2017**, *7*, 53-53, doi:10.1007/s13205-017-0682-0.
